# Application of time series and multivariate statistical models for water quality assessment and pollution source apportionment in an Urban River, New Jersey, USA

**DOI:** 10.1007/s11356-024-35330-2

**Published:** 2024-10-21

**Authors:** Oluwafemi Soetan, Jing Nie, Krishna Polius, Huan Feng

**Affiliations:** Department of Earth and Environmental Studies, Montclair State University, Montclair, NJ 07043 USA

**Keywords:** Lower Passaic River, Seasonal ARIMA, PMF model, Environmental monitoring, Water quality index

## Abstract

Water quality monitoring reveals changing trends in the environmental condition of aquatic systems, elucidates the prevailing factors impacting a water body, and facilitates science-backed policymaking. A 2020 hiatus in water quality data tracking in the Lower Passaic River (LPR), New Jersey, has created a 5-year information gap. To gain insight into the LPR water quality status during this lag period and ahead, water quality indices computed with 16-year historical data available for 12 physical, chemical, nutrient, and microbiological parameters were used to predict water quality between 2020 and 2025 using seasonal autoregressive moving average (ARIMA) models. Average water quality ranged from good to very poor (34 ≤ µWQI ≤ 95), with noticeable spatial and seasonal variations detected in the historical and predicted data. Pollution source tracking with the positive matrix factorization (PMF) model yielded significant *R*^2^ values (0.9 < *R*^2^ ≤ 1) for the input parameters and revealed four major LPR pollution factors, i.e., combined sewer systems, surface runoff, tide-influenced sediment resuspension, and industrial wastewater with pollution contribution rates of 23–30.2% in the upstream and downstream study areas. Significant correlation of toxic metals, nutrients, and sewage indicators suggest similarities in their sources.

**Graphical Abstract:**

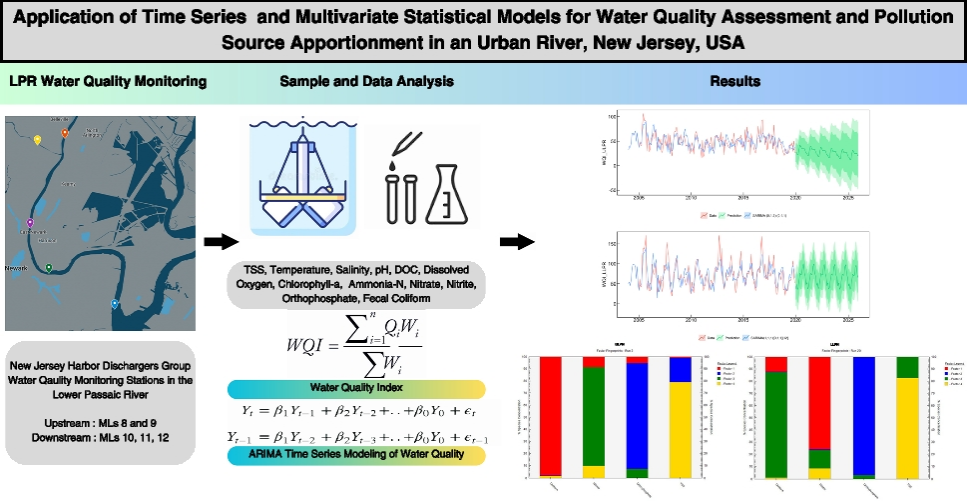

**Supplementary Information:**

The online version contains supplementary material available at 10.1007/s11356-024-35330-2.

## Introduction

Understanding and monitoring the integrity of water bodies worldwide is an overriding goal for environmental stakeholders as this plays a pivotal role in administering regulations, policies, and pollution mitigation or prevention approaches (Chapman & Sullivan 2022). Environmental monitoring has become very pertinent for freshwater and estuarine water bodies due to the growing intensity of pollution stress placed on them by human activities that facilitate point and non-point discharge of organic matter (sewage, domestic and industrial wastewater, leachate) and nutrients (sewage, fertilizer) into aquatic bodies. Currently, the crucial task of protecting and making restoration decisions in the water environment is enabled by applying remote sensing platforms and methods for quantitative estimation of water quality parameter concentrations, followed by computational and statistical assessments (Fu et al. 2023). The presence of these pollutants in global aquatic systems remains a serious issue due to their negative impacts on water quality, the hazard posed to aquatic ecosystems, and the disruption of human access to the resources provided by a water body (Manuel 2014). While nutrients occur naturally and are vital for aquatic ecosystems, unrestrained anthropogenic loading of these chemical substances leads to critical levels that engender eutrophication, increased oxygen demand, algae blooming, creation of aquatic dead zones, death of aquatic species, and destruction of existing balance in the ecosystems (Barletta et al. 2019). The introduction of sewage and organic matter into water bodies increases their pathogenicity and restricts recreational access to these water bodies, while also increasing the risk of human and animal mortality resulting from oral exposure to gastrointestinal and digestive disease-causing pathogens such as viral gastroenteritis, coliform, salmonella, and vibrio cholerae (Lin et al. 2022). Elevated levels of nitrogen and phosphorus in the water column can lead to algal blooms, and high levels of dissolved oxygen and ammonia–nitrogen in the water column are major contributors to water blackening and odor. Monitoring the changes in the concentration of these water quality parameters will provide a necessary indication of the polluted condition of the water environment (Fu et al. 2024). In highly urbanized rivers and estuaries where the potential for anthropogenic pollution is amplified owing to the proximity to human influence, water quality monitoring and pollution tracking are key for the development of effective pollution source control measures and remediation plans.

Consistent environmental data is very important for understanding the transitions of water quality in a specific study area from historical periods to the present (Levine et al. 2014; Mohammed et al. 2022). The analysis of this data can provide environmental stakeholders with the needed information on site characterization, site-specific regulatory measures, and the development of public guidance and advisories. Advanced information about future water quality tendencies can proactively help with developing environmental resilience and safeguarding measures to protect human and environmental health. Varying prediction modeling approaches including time series models, artificial neural networks machine learning models, and multivariate statistical models have been applied to predict missing information on water quality and forecast the future outlook of water bodies across the world (Abdul Wahid & Arunbabu 2022; Monteiro & Costa 2018; Setshedi et al. 2021). Specifically, the ARIMA time series model has been popularly used by several researchers to forecast water quality and quantity due to its resilience to seasonality and trend. Mombeni et al. (2013) validated their ARIMA model with historical data before predicting future water demand in Iran, while Hao et al. (2017), Graf (2018), and Tizro et al. (2014) all applied ARIMA modeling to the prediction of water quality parameters including pH, dissolved oxygen, temperature, nitrate, phosphate, salinity, and chlorophyll-a.

Pollution source tracking and apportionment is an important objective of environmental monitoring and data collection. Several source apportionment methods including multivariate dimensionality reduction methods, regression methods, chemical mass balance, and positive matrix factorization (PMF) have been used for the identification of pollution sources (Lv 2019). The PMF which is commonly associated with the US Environmental Protection Agency (USEPA) is a very powerful tool for air and water pollution source apportionment. This powerful multivariate statistical model was developed by Paatero and Tapper (1994) as a superior alternative to other multivariate analytical techniques due to its ability to explain the uncertainties normally associated with environmental data sampling and analysis. It has been widely applied for source apportionment of pollutants in water quality assessment (Proshad et al. 2023; Ren et al. 2023; Wu et al. 2023a, b). However, the PMF model is not without limitations. Other studies have reported increased errors in PMF predictions for input data from related sources (Frischmon & Hannigan 2024), rotational ambiguity in the PMF factor solutions such that the solutions are not unique, and variance in factor solutions for the same dataset based on how model parameters are set (Hemann et al. 2009).

The Lower Passaic River (LPR) has historically been polluted with sewage, toxic discharge from surrounding industrial establishments, stormwater depositions, and outputs from connecting rivers. Hence, the river components (sediments, biological tissue, and water) have been the subject of environmental investigations and research. Real-time sampling and statistical source analysis have been used to gauge water quality and identify the intensity and origin of pollutants (Iannuzzi et al. 2008; Onwueme & Feng 2006; Soetan et al. 2022). Although the public health risks of nutrient and sewage loading, as well as suspended solids concentrations in the river, have been evaluated and predicted (Jung 2017; Nie 2023), many gaps exist in the depth and robustness of our knowledge of water quality and pollution in the LPR, hence this study.

This study distinguishes itself from previous others because it employs a method of historical and robust water quality data analysis using a wide range of parameters, while also applying standard prediction and source apportionment models to achieve two important objectives: (i) provide useful and missing information on water quality during the environmental monitoring lag years and see further into the LPR water quality future (2020–2025) using time series modeling of the available 16-year data and (ii) determine the most probable sources of pollution into the LPR by using pollutant data as input for the PMF model and interpreting the outputs using previous studies and land use information. This study has the potential to provide future LPR research endeavors and stakeholders with reliable water quality information beyond the 5-year period which is missing in LPR databases, information on water quality trends in the river and pollutant source outlook which will enable policymakers to develop proactive and actionable plans for LPR pollution mitigation.

## Methodology

### Data and study area

Data was obtained for 12 physical and chemical parameters consisting of organic pollution indicators, nutrients, and physicochemical parameters including nitrate, nitrite, ammoniacal nitrogen (NH_3_-N), fecal coliform (F.coli), chlorophyll-a, orthophosphate (Ortho-P), dissolved organic carbon (DOC), temperature, dissolved oxygen (DO), pH, salinity, and total suspended solids (TSS). The dataset, extracted from the New Jersey Department of Environmental Protection (NJDEP) Data Miner database, was published by the New Jersey Harbor Dischargers Group (NJHDG) project and represents 16 years of environmental monitoring data from the long-term ambient water quality monitoring of the New Jersey portion of the NY/NJ Harbor waters. The purpose of this environmental monitoring project which spans several rivers, including the Elizabeth, Hackensack, Upper, and Lower Passaic, Pompton, Rahway, Raritan, and Saddle Rivers, is to document the baseline conditions of the aforementioned rivers and their changes over time, capture consistent data to facilitate tracking of temporal and external influences on water quality, and measure the influence of implemented pollution prevention and mitigation programs (GLEC 2015). The continuous nature of this data makes it very useful for understanding periodical water quality trends and also for predicting future water quality in the studied rivers. Monthly average data for the aforementioned parameters were extracted and used for water quality index computation for 2004–2019. The 16-year derived water quality index data was then used to predict water quality from 2020 to 2025, to account for missing water quality information and provide further future outlook. Additionally, 5 years of more recent data (2015–2019) was applied to a PMF model for pollution source investigation. Furthermore, toxic metal data from (Soetan et al. 2022) was extracted and correlated against water parameters to determine existing relationships.

**Fig. 1:**
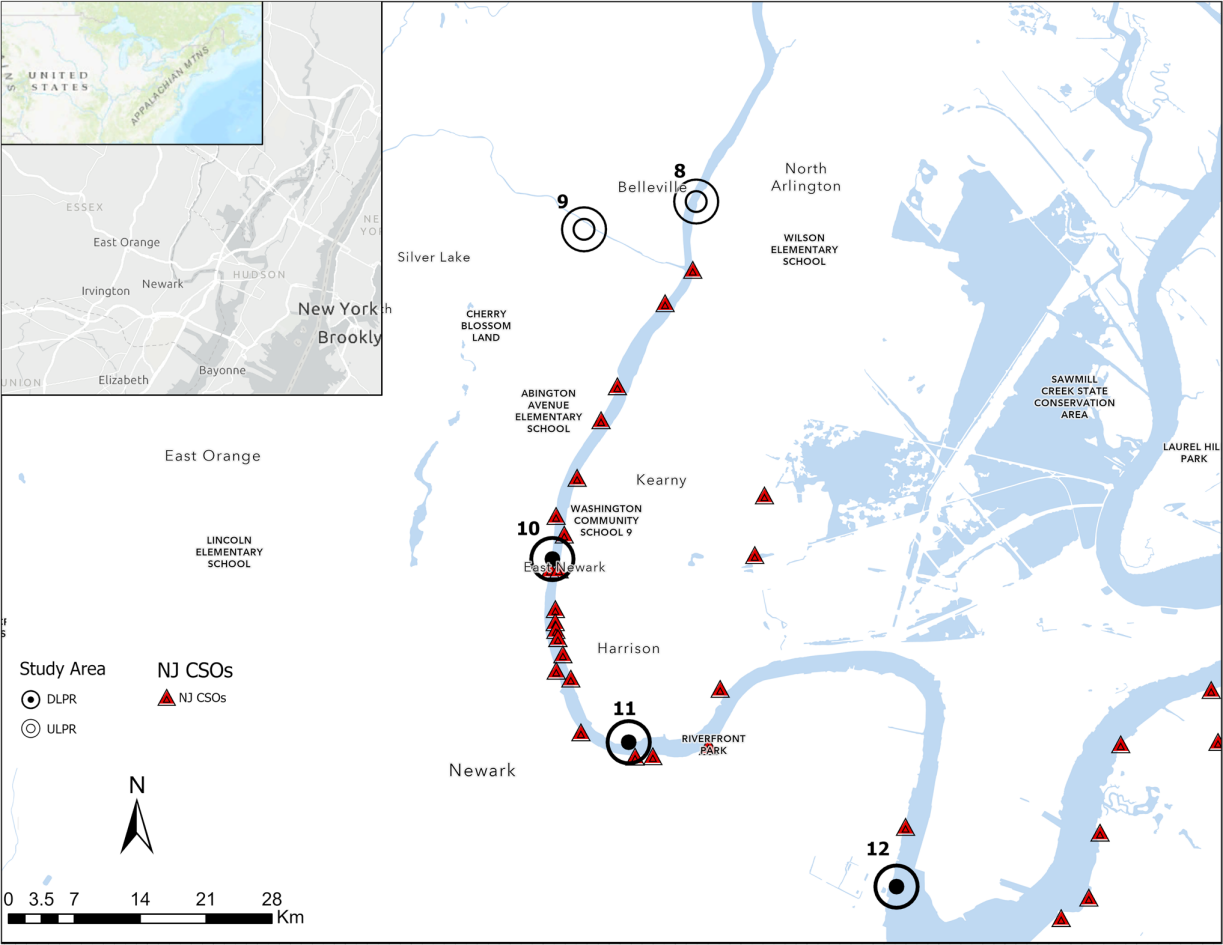
NJHGD monitoring locations (MLs) 8 and 9 represent the upstream study area ULPR, while MLs 10, 11, and 12 represent the downstream study area DLPR. ML 9 is situated in the Second River which is a major tributary of the LPR and its proximity to the LPR indicates that it significantly influences the water quality conditions in the river estuary. The distribution of combined sewer overflow (CSO) outfalls depicted shows greater CSO concentrations around MLs 10 and 11

The focus area of this study is the 13 km of the Lower Passaic River (LPR) which is located between 74°7′–74°10′ W and 40°42.9′–40°44.8′ N and forms a confluence with the Hackensack River into Newark Bay. Data was sourced from the five NJHDG monitoring locations (MLs), i.e., MLs 8, 9, 10, 11, and 12 within and around the area. ML 9 is located in the Second River but is very close to the LPR and is the only major tributary that directly discharges into this section of the Passaic River, significantly impacting water quality. As depicted in Fig. 1, the MLs are subdivided into two study areas based on their geographical proximity to Newark Bay as upstream LPR or ULPR (MLs 8 and 9) and downstream LPR or DLPR (MLs 10, 11, and 12). This estuarine river is heavily urbanized with anthropogenic pressure from human communities and industrial and coastal activities. The strategic positioning of the river also makes it a recipient of pollution outputs from upstream rivers including the Saddle, Pompton, Ramapo, Wanaque, and Pequannock rivers. The LPR area has been the subject of continuous environmental pollution investigations and remediation activities and is one of the four operable units of the Diamond Alkali Superfund site. An assessment of the water quality conditions in the area is necessitated by the continuously polluted status of the river, while a water quality forecast for the selected 6-year period (2020–2025) is warranted by a discontinuance of the NJHDG’s continuous environmental monitoring due to the COVID-19 pandemic, the consequential lack of available data for informing LPR environmental policy and management decisions.

### Water quality index

The water quality index (WQI) is a recognized tool used to assess the integrity of a water body based on pollutant and physicochemical properties concentrations measured in the study area. Several WQIs that have been developed and applied in past studies include the National Sanitation Foundation WQI, the Canadian Council of Ministers of the Environment WQI, Oregon WQI, and the Weighted Arithmetic WQI (Tyagi et al. 2020). For this study, the weighted arithmetic water quality index (WQI) was applied to assess the LPR water environment due to its ability to reflect the composite influence of different parameters on the water quality (Akoteyon et al. 2011). This WQI was developed by Brown et al. (1972) and classifies water quality according to the degree of purity and is calculated from the equation below:

1 $$\mathrm{WQI}= \frac{\sum {\mathrm{Q}}_{\mathrm{i}} \times {\mathrm{W}}_{\mathrm{i}}}{\sum {\mathrm{W}}_{\mathrm{i}}}$$

Q_i_ (the quality rating scale) and W_i_ (the parameter unit weight) are calculated using Eqs. 2 and 3:

2 $${\mathrm{Q}}_\mathrm{i}=100\left\lfloor\frac{{\mathrm{V}}_\mathrm{i}-{\mathrm{V}}_\mathrm{o}}{{\mathrm{S}}_\mathrm{i}-{\mathrm{V}}_\mathrm{o}}\right\rfloor$$

3 $${W}_{i}= \frac{K}{{\mathrm{S}}_{\mathrm{i}}}$$

*V*_i_ and *V*_o_ represent the measured and ideal concentration of parameter *i* (*V*_o_ = 0 for all parameters except pH (7.0) and DO (14.6 mgL^−1^)), *S*_i_ is the recommended guideline value of parameter *I*, and *K* is the proportionality constant calculated as.

4 $$K=\frac{1}{\sum \left(\frac{1}{{\mathrm{S}}_{\mathrm{i}}}\right)}$$

The WQI water quality rating ranges from excellent (WQI, 0–25) to unsafe/unsuitable (WQI, > 100) (Chauhan & Singh 2010; Uddin et al. 2021).

### Time series forecasting

Time series models are very useful analytical tools for predicting environmental conditions. Specifically, they have been prominently used for water quality prediction, with methods including ARIMA, simple exponential smoothing model (ETS), Facebook Prophet, and others being frequently applied in environmental studies in the literature (Kogekar et al. 2021; Abdul-Ahad and Subhee 2022; Sentas et al. 2018). The ARIMA model which is based on the Box-Jenkins approach (Box & Jenkins 1976) assumes that the data is non-seasonal and removes trends from the historical data provided to predict future scenarios with increased accuracy. The “AR” component of the model evidences the regression of the variable on its previous values, and the “I” component denotes how many times differencing has been performed to achieve trend stationarity, while the “MA” component shows that a linear combination of error values occurring at time intervals in the past make up the regression error (Dimri et al. 2020). To achieve a best-fitting model, a manual or automated iteration of the values of these components is required. For a non-seasonal ARIMA model (*p, d, q*), where *p* represents the lag order, *d* represents the order of differencing, while *q* denotes the order of moving average. The non-stationarity of the seasonal and multi-year data (2004–2019) in this study warrants the application of a seasonal ARIMA model which is differentiated from the ARIMA by the presence of additional autoregressive and moving average components, i.e., SARIMA (*p, d, q*) (*P, D, Q*)_*m*_ where *P*,* D*, and *Q* represent the AR, I, and MA for the seasonal component of the model, respectively, while *m* represents the number of periods in each season. The mathematical equations for the ARIMA and SARIMA models are given in Eqs. 5 and 6, respectively (Mombeni et al. 2013; Parmar & Bhardwaj 2014; Vagropoulos et al. 2016) as below:

5 $$\left(1- \sum_{\mathrm{i}=1}^{\mathrm{p}}{\upphi }_{\mathrm{i}}{\mathrm{L}}^{\mathrm{i}}\right) {\left(1-\mathrm{L}\right)}^{\mathrm{d}} {\mathrm{y}}_{\mathrm{t}}=\mathrm{c}+ \left(1+ \sum_{\mathrm{j}=1}^{\mathrm{q}}{\uptheta }_{\mathrm{j}}{\mathrm{L}}^{\mathrm{j}}\right) {\upvarepsilon }_{{\mathrm{t}}_{ARIMA}}$$

where *L*, ϕ_I_, and ε_tARIMA_ represent the lag operator, the moving average, and error terms respectively.

6 $${\upphi }_{\mathrm{p}}\left(\mathrm{B}\right){\Phi }_{\mathrm{P}}\left({\mathrm{B}}^{\mathrm{s}}\right){\left({1-\mathrm{B}}^{\mathrm{s}}\right)}^{\mathrm{D}}{\left(1-\mathrm{B}\right)}^{\mathrm{d}} {\mathrm{Z}}^{\mathrm{t}}= {\uptheta }_{\mathrm{q}}\left(\mathrm{B}\right) {\Theta }_{\mathrm{Q}}\left({\mathrm{B}}^{\mathrm{s}}\right) {\upvarepsilon }_{{\mathrm{t}}_{SARIMA}}$$

where ϕ_p_ (B) is the AR function of order p, Φ_P_ (B^s^) is the AR function of order P, θ_q_ (B) is the MA function of order q, while Θ_Q_ (B^s^) is the MA function of order Q. Also, Z^t^ is the forecast variable and ε_tSARIMA_ is the white noise, while (1-B)^d^ and (1-B)^D^ are the non-seasonal and seasonal differencing operators (Kogekar et al. 2021; Samal et al. 2019).

### Model selection and validation

To select the best-fit model for LPR WQI forecasting, different time series models were generated including an *auto-*SARIMA model, a SARIMA model generated by running several iterations of *p*,* d*,* q*, and *P*,* D*,* Q*, and an ETS model. The performances (fit) of these models and their forecasting accuracy were evaluated by static sample cross-validation. The 16-year data was divided into training (2004–2013) and test (2014–2019) datasets to achieve this. The models were fitted with the training set and cross-validated using the rolling window method which is similar to the k-fold method. The fixed-size training window was moved across the data and the model was trained and validated using subsequent year data. Cross-validation was useful for selecting the model with the best long-term prediction accuracy and reliability. The Akaike inclusion criterion (AIC), root mean square error (RMSE), mean absolute error (MAE), mean absolute percentage error (MAPE), and the Theil inequality coefficient metrics were used for model performance comparison. MAE is the average value of the absolute error or residual and is a function of the number of samples and the sum of absolute differences between the observed and predicted values. MAPE is the mean absolute percentage error, and lower values denote a higher prediction accuracy (Wu, et al. 2023a, b).

### Source apportionment

The USEPA-developed positive matrix factorization (PMF) model, which has been widely applied in pollution source allocation, was used to apportion organic pollutants in the study areas of the LPR. The PMF model is a very efficient multivariate statistical method that is based on factor analysis. It decomposes a sample and species concentration matrix (*A*) into a two-factor matrix of profiles (*B*) and contributions (*C*) and residual, *D* (Eq. 7).

7 $$A=B\times C+D$$

The best possible number of factors is obtained by repeated model runs to achieve several iterations and choosing the run with the best fit for the input data, i.e., the run with the least *Q*_robust_ value (Eq. 8).

8 $$Q= \sum_{\mathrm{i}=1}^{\mathrm{n}}\sum_{\mathrm{j}=1}^{\mathrm{m}}{\left(\frac{{e}_{\mathrm{ij}}}{{s}_{\mathrm{ij}}}\right)}^{2}$$

where *e*_ij_ represents the sum of squared differences between the original matrix (A) and the resultant PMF matrix (BC), while *s*_ij_ is the computed uncertainties.

The positive matrix factorization attempts to minimize the *Q* value for the profile and contributions while ensuring that only the least possible number of their components have negative values (Goswami & Kalamdhad 2022; Jiang et al. 2019).

To run PMF, two input data are required—the concentration data for the concerned parameters and the uncertainty data for the calculated or measured concentrations. For this study, the uncertainty is computed by modification of the method from Wang et al. (2016) such that where the minimum detection limit (MDL) is not provided, the standard deviation (SD) is used in its stead (Goswami & Kalamdhad 2022). When the parameter concentration was greater than the MDL or SD, Eq. 9 was applied; otherwise, uncertainty was computed using Eq. 10:

9 $$\mathrm{Uncertainty}= \frac{{\mathrm{C}}_{\mathrm{s}}}{10}+ \frac{\mathrm{MDL or SD }}{3}$$

10 $$\mathrm{Uncertainty}= \frac{5\left(\text{MDL or SD }\right)}{6}$$

To determine the optimal number of factors representing the ULPR and DLPR data, 100 iterations were run for different factor numbers between 3 and 6 under a random seed mode, to obtain the lowest possible *Q* value. Based on the *Q* and *R*^2^ values, the fit of the observed vs predicted scatter plot, and the bootstrap results, four factors representing the potential pollution sources were identified as optimal results for both the ULPR and DLPR study areas.

## Results and discussion

### Water quality in the Lower Passaic River

The annual statistics of water quality parameters over the 16 years (2004–2019) presented in Table S1 indicate that the average and maximum annual concentrations of water quality parameters in the ULPR, except for F.coli, were within the recommended aquatic life criteria (Table S2), although minor exceedances were recorded for the maximum values of DOC, nitrate, and chlorophyll-a. Similarly, in the DLPR, the recorded average concentrations of most parameters (except TSS and F.coli) were below the recommended criteria; however, the maximum values for TSS, DOC, orthophosphate, nitrite, F.coli, and chlorophyll-a exceeded the standard criteria. Although F.coli was comparably higher in the ULPR, other water quality parameters such as nutrients, TSS, DOC, salinity, and chlorophyll-a were higher in the DLPR. Also, DO concentrations indicate that oxygen availability for aquatic life was better in the ULPR than in the DLPR. These differences in water quality parameter concentrations are reflected in the annual average water quality index (WQI) computed for the two areas, with DLPR WQI exceeding ULPR WQI in all years between 2004 and 2019 by average exceedances of 19–88% (Table 1).

**Table 1:** Average annual water quality index (WQI) for the ULPR and DLPR from 2004 to 2019

Year	ULPRWQI		DLPRWQI		% difference (DLPR-ULPR)
	Value	WQI class	Value	WQI class	
2004	54	Poor	73	Poor	35
2005	64	Poor	87	Very poor	35
2006	52	Poor	65	Poor	24
2007	45	Good	81	Very poor	80
2008	54	Poor	65	Poor	19
2009	46	Good	63	Poor	36
2010	45	Good	71	Poor	58
2011	39	Good	54	Poor	39
2012	51	Poor	95	Very poor	88
2013	46	Good	71	Poor	54
2014	49	Good	72	Poor	48
2015	52	Poor	76	Very poor	45
2016	58	Poor	88	Very poor	51
2017	52	Poor	84	Very poor	61
2018	38	Good	54	Poor	42
2019	34	Good	56	Poor	66

Annual average water quality in the ULPR ranged from good (25 < WQI ≤ 50) to poor (50 < WQI ≤ 75) with 8 years of “good” water quality and 8 years of “poor” water quality recorded (Table 1). In the DLPR, the annual average water quality ranged from poor (50 < WQI ≤ 75) to very poor (75 < WQI ≤ 100). Neither of the study areas had “excellent” average annual water quality throughout the study timeframe, indicating that the full 13 km stretch of the LPR is subjected to pollution. Spatiotemporal differences in water quality indicate varying intensities of pollution across the upstream and downstream areas of the LPR. Results obtained from applying statistical recognition techniques in the historical, peer-reviewed literature on the LPR predominantly attribute the perpetuity of ecological risks and water quality degradation in the river to anthropogenic point and non-point sources (Israelsson et al. 2014). As an important aquatic ecosystem in the Passaic River watershed, the Lower Passaic River estuary conditions should remain optimal for aquatic life and meet the regional recommended aquatic life criteria (Ferreira et al. 2023); however, contrary to this, the LPR continues to be plagued by historical and persisting pollution, with organic and inorganic pollutants subjecting aquatic and human communities to enormous health and existential risks (Soetan et al. 2023). The continuous categorization of this stretch of the river as an operable unit of the Diamond Alkali Superfund site is evidence of sustained pollution in the river section. The worse status of water quality in the downstream LPR compared with the upstream areas highlights the presence of more contamination in this area. The DLPR has previously been the focus of contaminated sediment removal due to the historical direct discharge of toxic metals and organic pollutants from industrial establishments such as the Diamond Alkali “agent orange” facility (Romagnoli & Bonkoski 2012). Besides, while the full stretch of the 13 km area is impacted by tidal waves from Newark Bay, the proximity of the DLPR to Newark Bay and the constant confluence mixing with the Lower Hackensack River denotes that it is subjected to significantly greater turbulence caused by strong tidal waves which facilitate in situ pollution by unsettling sediment layers, resuspending particulate matter and releasing bound contaminants such as nutrients and other pollutants (toxic metals, dioxins, etc.) which may have been stored in sediments (Chen et al. 2018; Romero et al. 2019). Furthermore, as an estuarine environment, the retention of nutrients and other contaminants within the DLPR could be responsible for its poorer water quality status. Many studies have reported on the filtration and contaminant retention capacity of estuaries, finding that estuaries trap organic matter, sediments, and nutrients which may become available in the water column through turbulence, erosion, vertical mixing, and geochemical processes and thus increase TSS and water column nutrient concentrations (Chilton et al. 2021; López et al. 2021).

### Impacts of seasonality on water quality

**Fig. 2:**
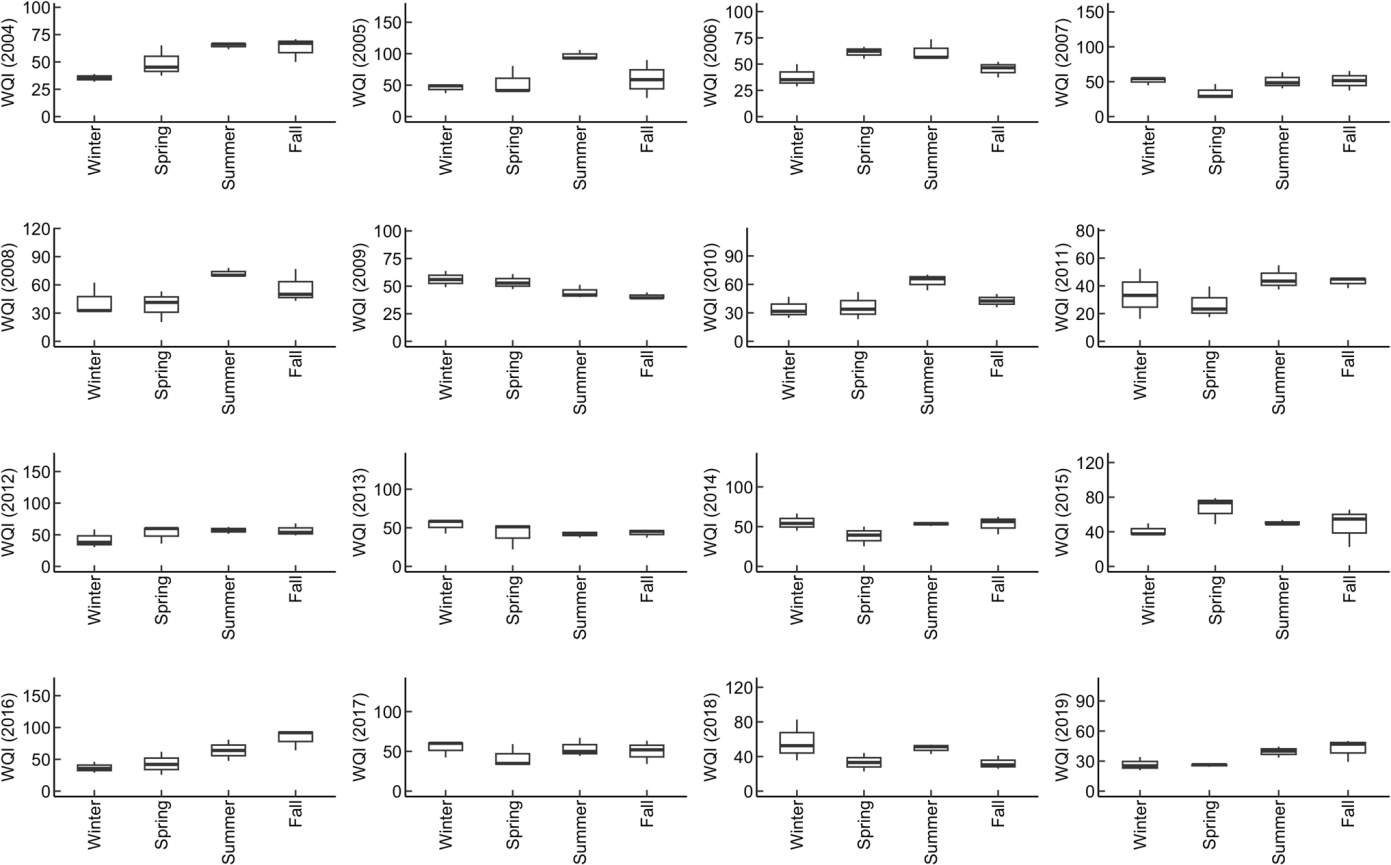
Seasonal changes in ULPR water quality (2004–2019): WQI ranged from “excellent” to “good” in the winter and spring but was generally poorer in the summer and fall

**Fig. 3:**
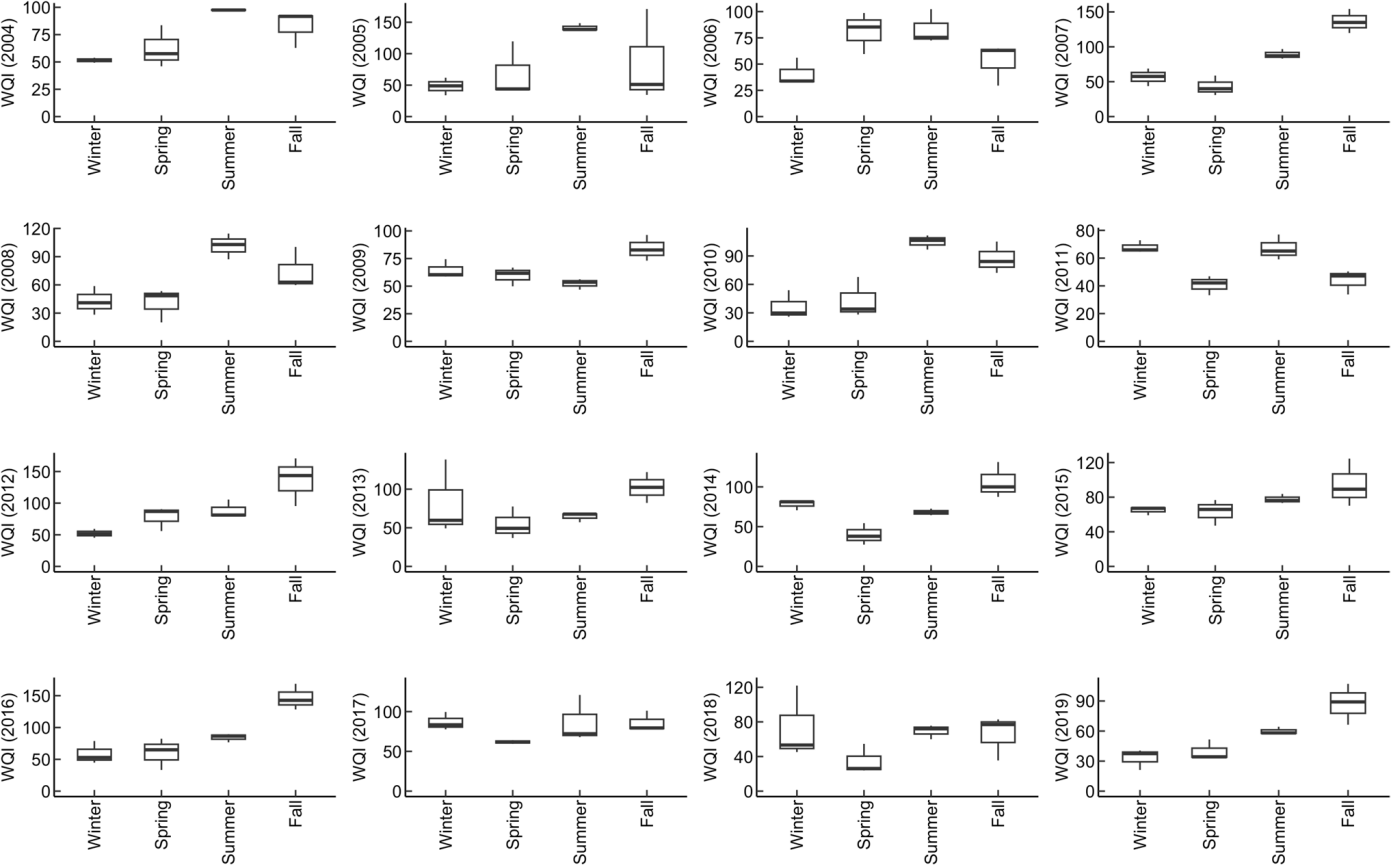
Seasonal changes in DLPR water quality (2004–2019): WQI ranged from “good” to “poor” in the winter and spring but was worse in the summer and fall with WQI ranging from “very poor” to “unfit for use”

**Fig. 4:**
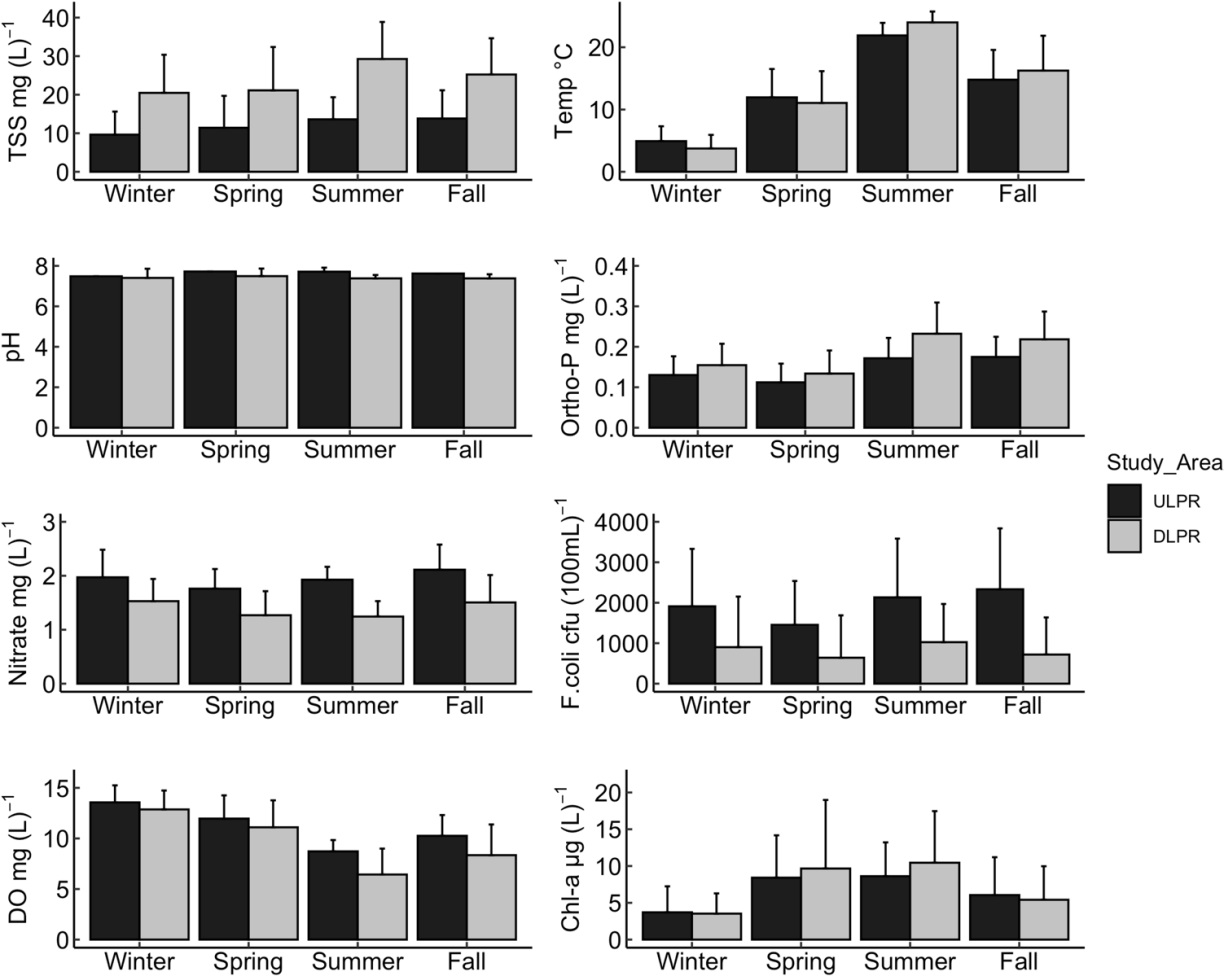
Seasonal 16-year average concentrations of water quality parameters in the ULPR and DLPR: temperature, TSS, and Ortho-P concentrations were noticeably higher in the summer and fall in both areas. In contrast, DO concentrations dropped as the seasons moved from winter to spring. Chlorophyll-a levels were distinctly higher in the spring and summer

Changes in water quality trends during the winter, spring, summer, and fall in both the ULPR and DLPR indicate a strong influence of seasonality on water quality in the Lower Passaic River. Generally, water quality was better in the winter and spring than during the summer and fall. For the ULPR, WQI ranged from “good” (27, 26) to “poor” (56, 67) in the winter and spring seasons, respectively, and from “good” (29, 35) to “very poor” (76, 93) in the summer and fall seasons, respectively (Fig. 2). In the DLPR, WQI ranged from “good” (33, 35) to “very poor” (87, 81) in the winter and spring, respectively, and from “good” (52, 44) to “unfit for use” (142, 147) in the summer and fall seasons, respectively (Fig. 3). The influence of seasonality on water quality parameters was mostly evident in Ortho-P, chlorophyll-a, F.coli, DO, and TSS (Fig. 4). Average concentrations of TSS, Ortho-P, and F.coli were greater in the summer and fall for both study areas, but in contrast, DO was higher in the winter and spring, while chlorophyll-a was higher in the spring and summer. The highest and most consistent exceedance of the water quality criteria (235–1650% in the ULPR and 55–723% in the DLPR) was recorded for F.coli. The heavy rainfall events that characterize the summer and fall months in the LPR can be identified as an important factor responsible for poorer water quality during these seasons due to the resulting increase in surface runoff and combined sewer overflow events (Jung 2017). Additionally, increases in temperature during the summer and fall (Fig. 4) amplify the evaporation of water from the river and reduce the mixing and dilution of pollutants due to decreased water volumes (Xia et al. 2016). This effect is especially telling in the summer season where elevated temperatures are not accompanied by rainfall events, which thus results in relatively high nutrient loads that elevate the risks of eutrophication and also increase biological and chemical oxygen demand, thus reducing oxygen availability for aquatic life and facilitating algae proliferation (Mu et al. 2017).

Chlorophyll-a is an indicator of algal and phytoplankton levels in a water body. Its trend as shown in Fig. 4 depicts an increase from winter to spring as the temperature rises and a decrease from summer to fall as temperature drops. This trend is similar to what has been reported in other studies, where phytoplankton growth and proliferation in aquatic bodies are sensitive to temperature rises due to the warmness of water and increased light penetration that facilitates their growth (Ho & Michalak 2020; Lyngsgaard et al. 2017). The combined negative effect of high temperature and nutrient pollution on aquatic life’s access to oxygen is most telling in the summer as the river water becomes warm and oxygen solubility decreases in the water, with organic matter-decomposing microbes further lowering the amount of oxygen available for aquatic organisms (Chapra et al. 2021). The high TSS levels recorded during the summer and fall can be attributed to turbulence caused by increased storm events, surface runoff, and sediment transport from construction activities that characterize the summer, waste discharge, in situ particle resuspension and erosion, and an increase in phytoplankton proliferation (Mamun et al. 2022).

The estuarine DLPR is at the mouth of the LPR and has increased water depth and volume and is thus more strongly impacted by tidal exchanges with Newark Bay due to the direct relationship between water depth and tide speed (HydroQual Inc., 2008). These tidal influences are further amplified during high-flow events influenced by heavy rainfall events. Estuarine environments like the DLPR are especially vulnerable to the effect of tidal changes—they experience high turbulence and material resuspension due to tidal currents, and this causes the release of buried nutrients and pollutants, increasing their bioavailability to estuaries’ rich aquatic biota and elevating their concentrations in the water column (Devlin & Pan 2020; Khojasteh et al. 2023).

### Water quality forecasting

Initial analyses of the ULPR and DLPR time series data with autocorrelation (ACF) and partial autocorrelation (PACF) functions are depicted in Figs. S1 and S2. The ACF plots show a cyclical nature with significant peaks at biannual or annual periods, an indication of seasonality and trend in the data. Further stationarity and seasonality tests with the augmented Dickey- and Olleck-Webel tests confirmed the conclusions of non-stationarity and seasonality made for the data using the ACF/PACF plots. The fit and forecasting accuracies of an automated SARIMA model, an iterated SARIMA model, and an ETS model were evaluated through a comparison of AIC, BIC, RMSE, MAPE, MAE, and MASE metrics (Table 2). These parameters are important criteria for evaluating the prediction accuracy of the model and how well it fits the data that it was generated from, with lower values suggesting a better model (Uddin et al. 2023).

**Table 2:** Comparison of model fit statistics and accuracy metrics for SARIMA and ETS generated models for the ULPR and DLPR study areas

				Model fit statistics		Model accuracy metrics					Ljung-Box test			
Study area	Model	Order	Seasonal order	AIC	BIC	RMSE	MAE	MASE	MAPE	Theil coefficient	Statistics	DF	Sig	No of outliers
ULPR	SARIMA	(0,1,2)	(0,1,1)_12_	1491	1504	16.6	12.7	0.8	22.9	0.8	16.3	21	0.75	0
		(1,1,1)	(0,1,1)_12_	1489	1502	19.3	14.8	0.9	25.2	1.0	14.3	21	0.86	0
	ETS	-	-	2039	2088	15.0	11.8	0.7	22.2	0.8	21.0	10	0.02	0
DLPR	SARIMA	(0,1,2)	(2,1,1)_12_	1654	1673	35.8	29.4	0.9	55.1	1.4	16.8	19	0.60	0
		(1,1,1)	(0,1,1)_12_	1655	1668	25.3	19.5	0.6	31.2	0.8	20.3	21	0.50	0
	ETS	-	-	2214	2269	270.9	234.5	7.0	428.5	11.5	46.0	8	0.00	0

The statistics obtained for the fitted models and the accuracy metrics recorded during cross-validation of the models by prediction of test data indicate that the SARIMA(0,1,2),(0,1,1)_12_ model is the most adequate for forecasting water quality in the ULPR, while the SARIMA (1,1,1), (0,1,1)_12_ model is the best fit for DLPR water quality prediction. Although the ETS model fitted to the ULPR input data performed better during cross-validation, it had a comparably higher AIC than the SARIMA models—an indication of its poor *goodness of fit* to the data (Table 2). Further evaluation with the Ljung-Box test showed strong evidence that the ETS model lacked a good fit to the data due to persisting autocorrelations (*p* < 0.05); however, the selected SARIMA models displayed a good fit to the residuals (*p* > 0.05). The trend and cyclical nature of the time series further validate the selection of a seasonal ARIMA model, since it is resilient towards data seasonality and has an excellent prediction capacity for data with trend features (Wu, et al. 2023a, b).

**Fig. 5:**
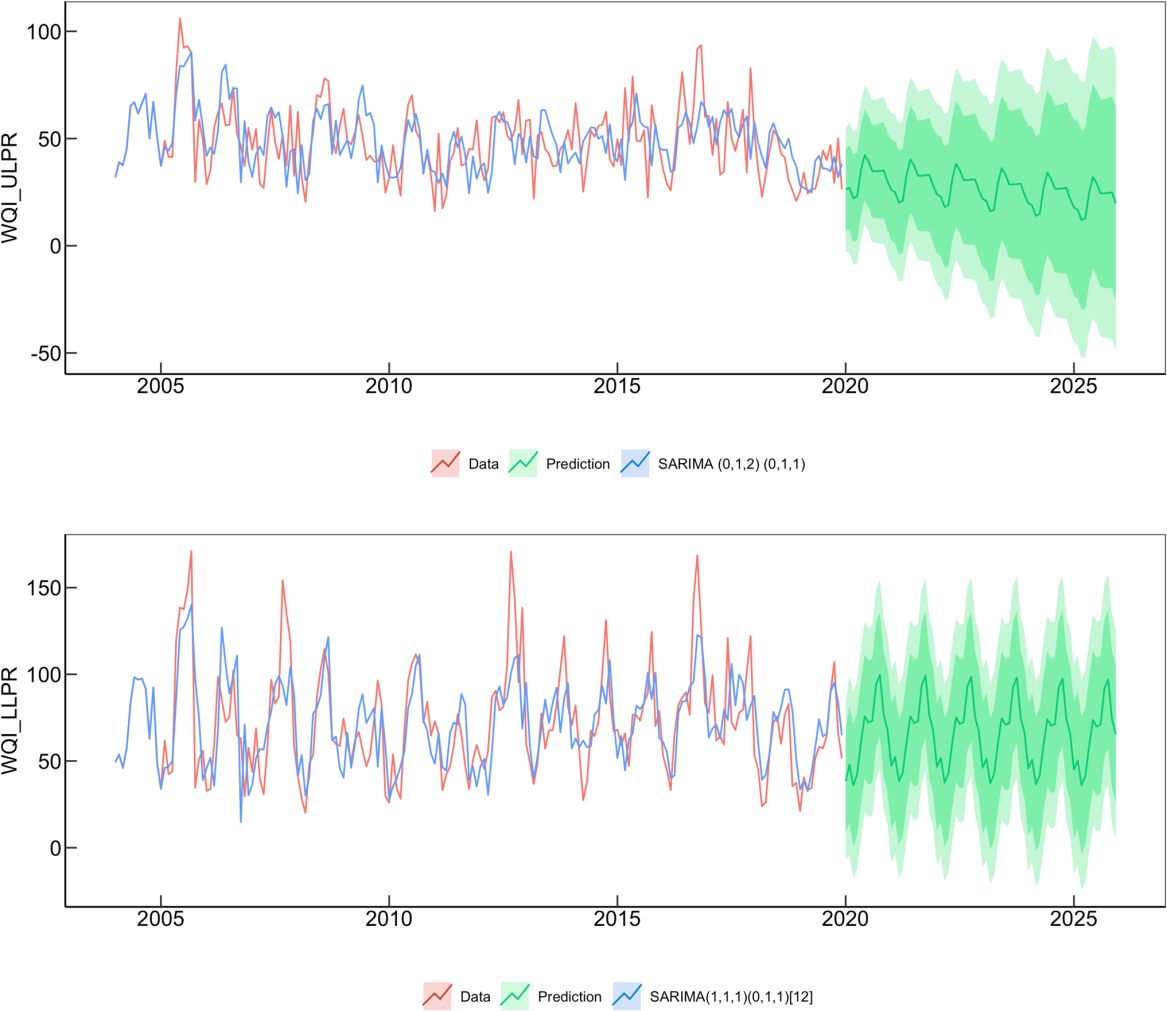
SARIMA (0,1,2) (0,1,1) and (1,1,1) (0,1,1) models are fitted to the observed data and 6-year forecast of WQI in the ULPR and DLPR, respectively. Seasonality and trend are evident in the WQI graphs, while water quality was better in the ULPR than the DLPR in predicted values

The 6-year forecast results (Table S3) revealed a continuity in previously established water quality trends with predicted water quality in the ULPR (Fig. 5a) significantly better than the water quality predicted for the DLPR (Fig. 5b). The seasonality earlier detected in the data was noticeable in the predicted data (Table 3) with average winter water quality better than the spring—ranging from excellent in the ULPR (20, 28) to poor in the DLPR (43, 56)—than in the summer and fall where it ranged from good in the ULPR (28, 40) to very poor in the DLPR (72, 90).

**Table 3:** Point forecast values for WQI in the ULPR and DLPR study areas using SARIMA (0,1,2) (0,1,1), and SARIMA (1,1,1) (0,1,1), respectively

Year	2020				2021				2022				2023				2024				2025			
Season	Winter	Spring	Summer	Fall	Winter	Spring	Summer	Fall	Winter	Spring	Summer	Fall	Winter	Spring	Summer	Fall	Winter	Spring	Summer	Fall	Winter	Spring	Summer	Fall
ULPR	26.6	26.8	38.8	34.9	27.0	24.7	36.7	32.9	21.6	22.7	34.7	30.8	22.9	20.6	32.6	28.8	20.8	18.6	30.6	26.7	18.8	16.5	28.5	24.7
DLPR	43.0	46.2	73.7	90.2	55.8	47.5	73.5	89.7	55.3	46.9	73.0	89.2	54.8	46.4	72.5	88.7	54.2	45.9	71.9	88.1	53.7	45.4	71.4	87.6

### Pollution source apportionment

**Fig. 6:**
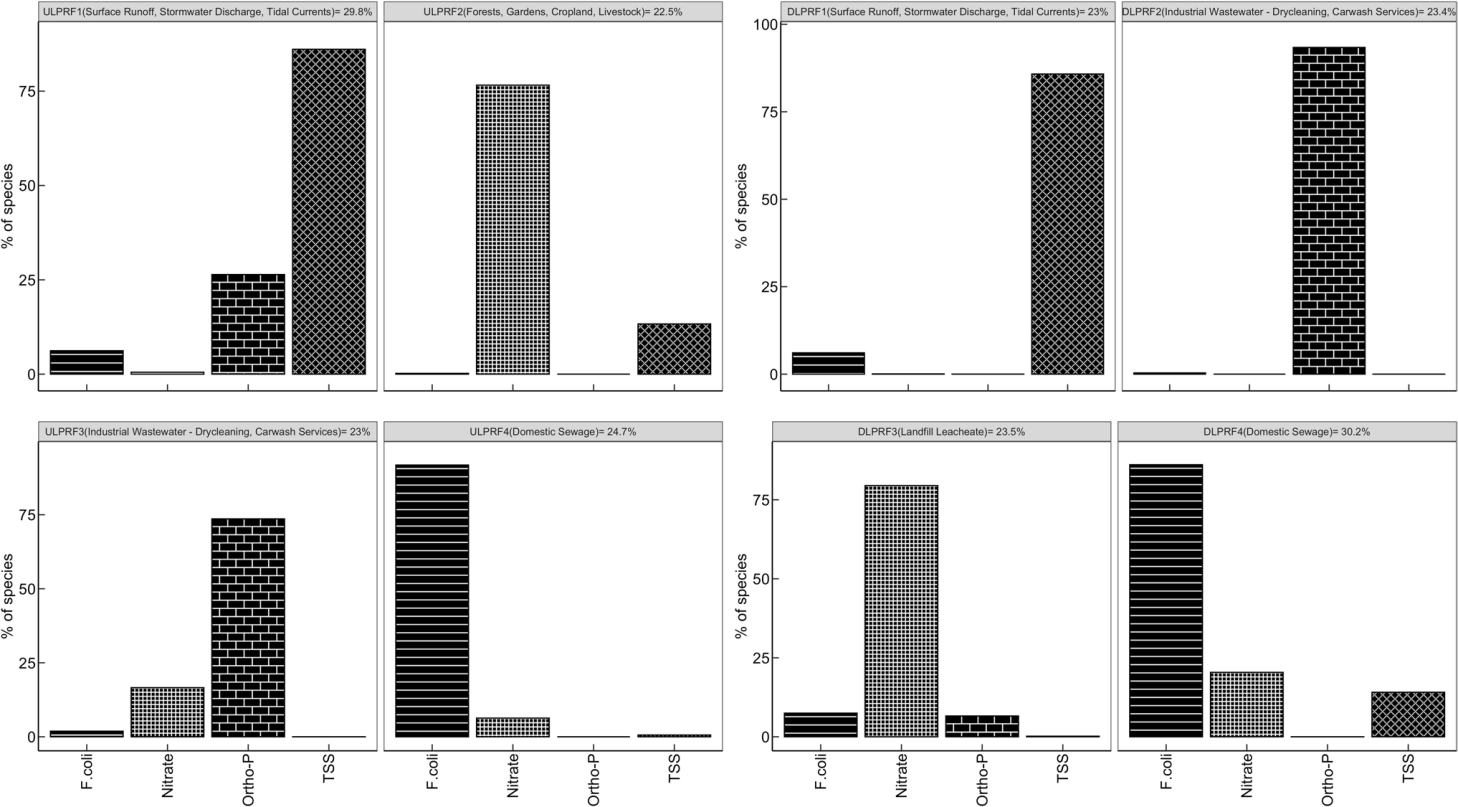
The percentage contribution of water quality parameters to each identified source in the ULPR and DLPR. The PMF model identified a four-factor solution as the optimal number of factors for each study area. Percentage pollution contribution of each identified factor ranged from 23 to 30%

The PMF model results for both study areas indicate that the water quality indicators (F.coli, nitrate, orthophosphate, and TSS) exhibited excellent fit upon implementing the 4-factor solution. The optimal number of factors gives the lowest *Q*_robust_/*Q*_true_ value and the highest parameter *R*^2^ values, indicating a good fit between the observed and predicted data (Y. Li et al. 2020a, b). The *R*^2^ value for all the water quality indicators in the DLPR and ULPR ranged from 0.99 to 1 (Table S4), denoting a strong correlation between the predicted and observed data and underscoring the accuracy of source apportionment carried out by the PMF. In Fig. 6, the PMF model results depict the contribution of each water quality indicator to the derived factors (identified sources). The contribution rate of each of the identified factors to the total source contribution is calculated from the PMF base outputs, and these rates ranged from 23 to 29.8% in the ULPR and 23 to 30.2% in the DLPR. The identified source for each factor is determined by the dominant parameters within the factor and the pre-existing knowledge that the Lower Passaic River area is predominantly urbanized, with industrial activities, human residence, and open lands dominating the bulk of land use (Du et al. 2018). Factor 1 (ULPRF1 and DLPRF1) are both dominated by TSS (86.1%, 85.8%), with Ortho-P (26.4%), and F.coli (6.2%) as the other main loading parameters in ULPRF1 while F.coli (6.1%) is the only other main loading parameter in DLPRF1. These factors can be attributed to surface runoff and stormwater discharge resulting from rainfall events. Stormwater loosens and transports particulate material and other pollutants, discharging them into rivers as runoff or through stormwater channels (He et al. 2019). Also, depending on the intensity of storm events, the river current may be increased such that the flow rate is strong enough to overcome the binding and cohesive energy of bedded sediments, suspending them and increasing the water turbidity (Lu et al. 2018). Marine traffic, offshore construction activities such as dredging, and offshore road and infrastructure construction activities are rife around the LPR, and these activities significantly contribute to TSS levels. The sediment load generated from onshore construction activities degrades water quality by transporting other contaminants into the water and increasing river turbidity (Zhu et al. 2023). Additionally, offshore construction activities and marine traffic as well as strong tidal currents and waves particularly in the DLPR potentially unsettle and cause the vertical flux of particulate matter from the sediment bed, increasing water turbidity and suspended particulate load, and releasing nutrients, and pollutants that were previously buried in the sediments (Paudel et al. 2019). These factors (ULPRF1 and DLPRF1) account for 29.8% and 23% of the total source contributions in the ULPR and DLPR, respectively. Factor 2 in the ULPR (ULPRF2) and Factor 3 in the DLPR (DLPRF3) are both dominated by nitrate (76.6%, 79.5%) with TSS (13.3%) as the other main loading parameter in ULPRF2 and F.coli (7.5%) and Ortho-P (6.6%) as the other main loading parameters in DLPRF3. The domination of ULPRF2 by nitrate and the significant contribution of TSS suggests that this source can be attributed to agricultural and yard fertilizer application, forest-related runoff, animal manure, and livestock feedlots in the surrounding area. While agriculture is not a dominant source of land use in the ULPR area, forests and human habitation form a significant part of the upstream land use (Nie 2023); thus, fertilizer runoff from yard and lawn maintenance and decayed organic matter from forests and livestock could be the source of nitrate. Nitrate dominance in DLPRF3 can be traced to the seepage of leachate and decaying organic matter from landfills, which would also explain the significance of phosphate and F.coli. The 94-acre Meadowland Landfill around the Newark and Hackensack areas discharges an estimated 83,000 gallons of leachate waste into the DLPR daily causing organic pollution and increasing the toxic load of heavy metals which are contaminants of serious concern for the LPR (Bock et al. 2021). These factors represent 22.5% and 23.5% of the total ULPR and DLPR total source contributions, respectively. Factor 3 in the ULPR (ULPRF3) and Factor 2 in the DLPR (DLPRF2) are both dominated by Ortho-P (76.6%, 93.4%) with nitrate (16.6%) as the only other main loading parameter. These factors can be attributed to industrial wastewater discharge into the Lower Passaic River. Nie (2023) outlined the presence of several dry-cleaning services across the Lower Passaic River. Phosphates are an integral component of cleaning detergents as they break down dirt and prevent it from reabsorbing to material surfaces (Chen et al. 2022).These factors account for 23% and 23.4% of the ULPR and DLPR total source contributions. Factors 4 in both the ULPR (ULPRF4) and DLPR (DLPRF4) are dominated by F.coli (91.8%, 86.1%), with nitrate (6.3%) as the other main loading parameter in ULPRF4 and nitrate (20.4%) and TSS (14.1%) as the other main loading parameters in DLPRF4. Factor 4 in the two areas indicates domestic sewage as the most probable pollution source. Figure 1 depicts the spread and intensity of combined sewer system outfalls in the Lower Passaic River. These outfalls represent about 25% of New Jersey’s total CSS outfalls and account for untreated and partially treated sewage, stormwater runoff, and industrial wastewater (Jung 2017; Soetan et al. 2022). These factors account for 24.7% and 30.2% of the total source contributions in the ULPR and DLPR, respectively. The domination of fecal pollution among pollution sources in the DLPR can be attributed to the intensity of CSO outfalls in the study area. Figure 1 shows that the majority of CSO outfalls in LPR lie between MLs 10–12. Soetan et al. (2022) noted the potential of these CSOs to constantly overflow even with very light rainfall (< 0.1 inch/h) over a short period. There are a lesser number of CSO outfalls in the ULPR area; however, it remains within the influence of tidal waves, and this fecal contamination in the ULPR can be associated with tidal upstream transport of sewage from the DLPR to the ULPR, as well as seepage of sewage from septic tanks and livestock waste in the surrounding area.

### Correlation of physicochemical parameters with toxic metals in the LPR

**Fig. 7:**
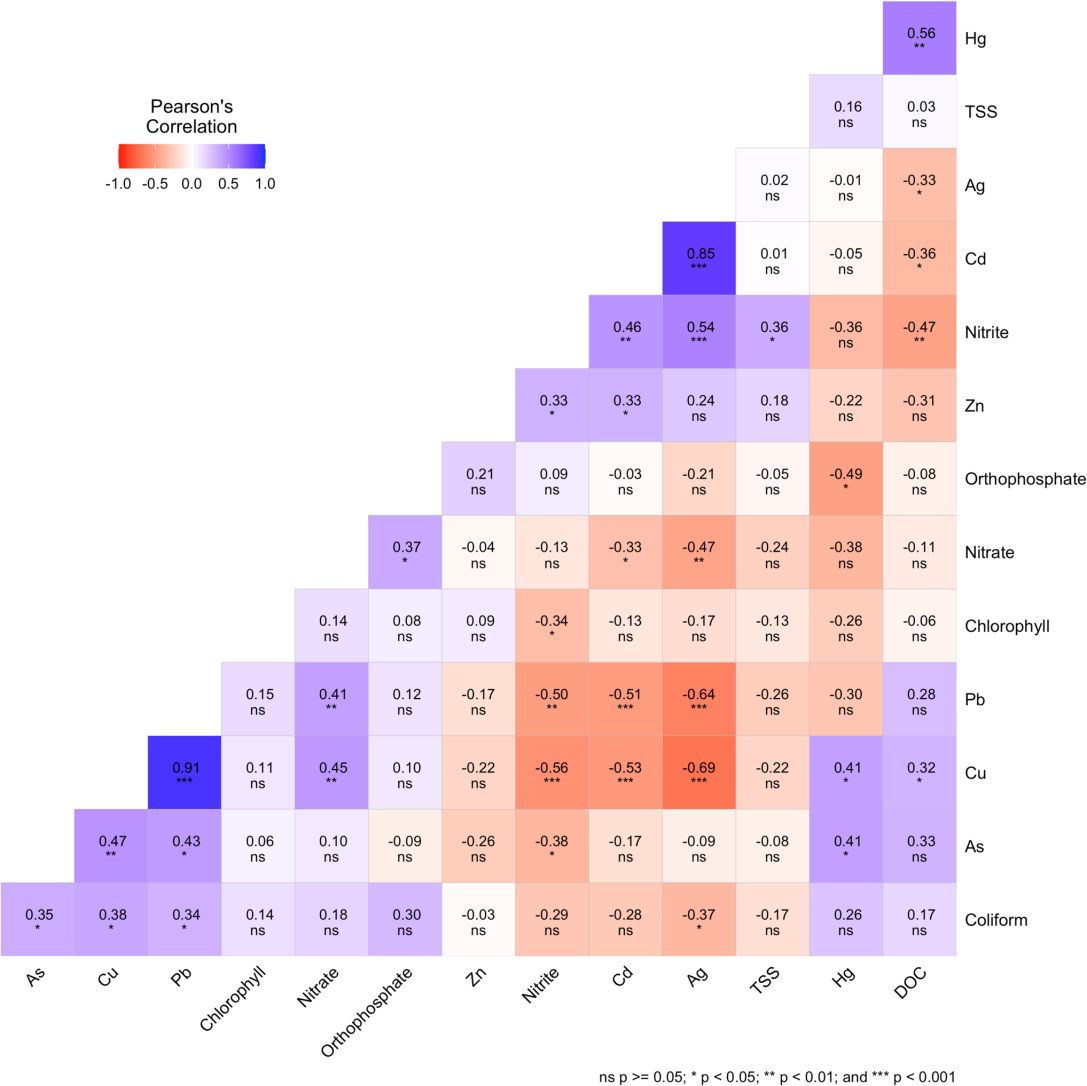
Pearson correlation analysis of physicochemical parameters with toxic metals in LPR sediments indicating potential pollution-source relationships

Previous studies have investigated toxic metal contamination and ecotoxicological impact on the Lower Passaic River (Jung 2017; Soetan et al. 2022). To gain a further understanding of potential source and effect relationships between the toxic metals and water quality indicators evaluated in this study, metal and water quality data were analyzed using the Pearson correlation method. Figure 7 shows the existing associations between sedimentary toxic metals and the physicochemical parameters.

The significant (*p* < 0.05) positive relationships that were recorded between silver (Ag), cadmium (Cd), zinc (Zn), and nitrite, as well as between copper (Cu), lead (Pb), and nitrate and also between arsenic (As), Cu, Pb, and coliform, are potentially indicative of the similarities in anthropogenic sources. Silver nitrate and nanoparticles can be found in landfills and other hazardous waste sites, sewage outfalls, as waste from photo-processing industries, and also used in wastewater treatment plants for microbiological water treatment (Forstner et al. 2020; Kolesnikov et al. 2020). Manure and fertilizers used in agricultural croplands, lawns, and gardens have been identified as potential sources of Cd, Cu, Pb, and Zn runoff into water bodies. Moreover, nitrogenous fertilizers have specifically been reported to influence the solubility and availability of these metals (Wei et al. 2020; Xu et al. 2020). Leachate from landfills has been reported to contain high concentrations of toxic metals and nitrogen compounds. Agricultural fertilizers, pesticides, and manures are other potential sources of these contaminants (Li et al. 2020a, b; Panagos et al. 2018). The association of As, Cu, and Pb with F.coli is potentially a result of similar output from combined sewer overflows (CSOs) that discharge stormwater, industrial wastewater, and municipal sewage into the Lower Passaic River. The metals are associated with road dust and vehicular discharge which are released into CSOs and stormwater sewers when surface runoff loosens surface and subsurface soils (Hwang et al. 2016; Liu et al. 2019). The permitted discharge of industrial effluent into combined sewer systems is also another route for the metals into the aquatic system and may account for their association with F.coli (Rouff et al. 2013).

The significant association between Hg and DOC has been attributed to the existence of a coupling relationship between the two, resulting from similar transportation and transformation processes, with increases in aquatic DOC, indicating a corresponding increase in Hg levels (Lavoie et al. 2018). Similarly, a positive, significant correlation between Cu and DOC is indicative of the existence of a strong interaction. The solubility and toxicity of Cu are very dependent on DOC, with strong covalent complexation reactions between the two reducing Cu toxicity and bioavailability in the aquatic environment (Filipović et al. 2023; Zitoun et al. 2019).

## Limitations of the study

This study successfully applies water quality and statistical analysis models to predict water quality and pollution sources but has limitations relevant to its direct application for policymaking. SARIMA models yield reliable predictions but are less accurate than some machine learning (ML) models. Future research should use precise, scalable ML models like artificial neural networks (ANN) and random forests (RF) for better data quality and policy effectiveness. Additionally, inconsistency in the applied methodology (detection limit and standard deviation method) for computing uncertainty input data for the PMF model might have affected the results. The study also overlooks factors like climate change, policy changes, and land use changes that impact future water quality. Future research must include these future forcing conditions and assess input assumptions and errors using sensitivity assessments, Monte Carlo simulations, and generalized likelihood uncertainty estimations.

## Conclusion

The time series prediction of water quality in the LPR revealed the prevailing conditions during a 5-year lag period in environmental monitoring data availability. Spatiotemporal water quality analysis indicates significant variation in water quality between the upstream (ULPR) and the downstream (DLPR) Lower Passaic River study areas, with ULPR consistently maintaining better water quality throughout the recorded periods (34 ≤ µWQI ≤ 64), while water quality was worse in the DLPR (54 ≤ µWQI ≤ 95). Seasonal variation was noticeable in the data with better water quality in the spring and winter compared with the summer and fall, where water quality regression is evidenced by pronounced changes in dissolved oxygen, nutrients, and total suspended solids concentrations.

Combined sewer overflows and surface runoff were identified as similar pollution sources for both the upstream and downstream reaches of the LPR based on land use information and previous studies indicating the existence of intense urbanization and high population density in the area. Sediment resuspension in the DLPR estuarine area is suspected to be responsible for a significant portion of pollution in the area especially as it is a pollutant repository that is constantly influenced by strong tidal waves from Newark Bay.

Water quality prediction showed that particular attention should be paid to the DLPR, and it should be the priority of environmental mitigation efforts due to the existing ecological threats from anthropogenic and in situ pollution. It is recommended that further studies and environmental monitoring projects incorporate other pollutants such as toxic metals, PCBs, dioxins, and PAHs which have been identified for the Lower Passaic River.

## Supplementary Information

Below is the link to the electronic supplementary material.

Supplementary file1 (DOCX 293 KB)

## Data Availability

Data used in this study was obtained from the publicly accessible NJ Data Miner database.
