# Supplementary material for: Application of time series and multivariate statistical models for water quality assessment and pollution source apportionment in an Urban River, New Jersey, USA
Source: Environ Sci Pollut Res Int. 2024 Oct 21;31(52):61643–59. doi: 10.1007/s11356-024-35330-2 (PMC11541290; doi:10.1007/s11356-024-35330-2)
Supplement: Supplementary file 1 — Supplementary file1 (DOCX 293 KB) [file 11356_2024_35330_MOESM1_ESM.docx]

**Supplementary Information**

**Application of Time Series and Multivariate Statistical Models for Water Quality Assessment and Pollution Source Apportionment in an Urban River, New Jersey, USA**

Oluwafemi Soetan^1^, Jing Nie^1^, Krishna Polius^1^, Huan Feng^1*^

^1^ Department of Earth and Environmental Studies, Montclair State University, Montclair, NJ 07043, USA

^*^ Corresponding author: Huan Feng (Email: [fengh@montclair.edu](mailto:fengh@montclair.edu))

| Table S1 Statistics of water quality parameters in the ULPR and DLPR through 16 years (2004 - 2019) | | | | | | | | | | | | | | | | | | | | | | | | | |  |
| --- | --- | --- | --- | --- | --- | --- | --- | --- | --- | --- | --- | --- | --- | --- | --- | --- | --- | --- | --- | --- | --- | --- | --- | --- | --- | --- |
| Year |  | TSS mgL^-1^ | | DOC mgL^-1^ | | Temp °C | | Salinity ppth | | pH |  | Ortho-P  mgL^-1^ | | Nitrite  mgL^-1^ | | Nitrate  mgL^-1^ | | NH_3_-N  mgL^-1^ | | F.Coli  cfu 100mL^-1^ | | DO  mgL^-1^ | | Chl a  µgL^-1^ | |  |
|  |  |  |  |  |  |  |  |  |  |  |  |  |  |  |  |  |  |  |  |  |  |  |  |  |  |  |
| *Site* |  | a | b | a | b | a | b | a | b | a | b | a | b | a | b | a | b | a | b | a | b | a | b | a | b |  |
| 2004 | Avg. | 9 | 18 | 5.4 | 6.3 | 13 | 13.5 | 0.3 | 4 | 7.3 | 7.1 | 0.2 | 0.2 | 0.03 | 0.04 | 2.1 | 1.5 | 0.18 | 0.2 | 767 | 654 | 10 | 9 | 3.4 | 6.8 |  |
|  | SD | 5 | 7 | 1.6 | 1.8 | 6.6 | 8.2 | 0.1 | 2.2 | 0.5 | 0.5 | 0.1 | 0.1 | 0.01 | 0.01 | 0.2 | 0.3 | 0.03 | 0.04 | 440 | 645 | 2 | 3 | 2.4 | 5.3 |  |
|  | Min | 2 | 5 | 2.8 | 3 | 4 | 1.4 | 0.1 | 0.4 | 6.3 | 6.1 | 0.1 | 0.1 | 0.02 | 0.02 | 1.7 | 1 | 0.09 | 0.14 | 70 | 35 | 7 | 5 | 0.8 | 1.8 |  |
|  | Max | 15 | 33 | 8.3 | 9.5 | 22.1 | 23.5 | 0.4 | 6.9 | 8 | 7.9 | 0.3 | 0.3 | 0.04 | 0.05 | 2.6 | 1.9 | 0.19 | 0.3 | 1394 | 2362 | 14 | 14 | 7.9 | 15.2 |  |
|  | Kurt | -1 | 2 | -0.2 | 0.1 | -1.6 | -1.6 | 3.3 | -1.2 | -0.3 | 0.3 | -0.7 | -1.3 | -1.49 | -1.9 | 1.3 | 0.2 | 12 | 3.23 | -1 | 4 | -2 | -1 | -0.2 | -1.4 |  |
|  | Skew | 0 | 0 | 0 | -0.1 | 0 | -0.1 | -1.3 | -0.2 | -0.2 | -0.5 | -0.2 | -0.1 | 0 | -0.17 | 0.2 | -0.2 | -3.46 | 1.32 | 0 | 2 | 0 | 0 | 1 | 0.6 |  |
| 2005 | Avg. | 12 | 22 | 3.2 | 4.8 | 13.7 | 13.7 | 0.3 | 2.8 | 7.4 | 7.3 | 0.2 | 0.3 | 0.03 | 0.05 | 2 | 1.6 | 0.24 | 0.24 | 957 | 396 | 12 | 12 | 8.6 | 9.5 |  |
|  | SD | 7 | 12 | 2.3 | 4.3 | 8.1 | 9.5 | 0.1 | 3.2 | 0.4 | 0.3 | 0.1 | 0.1 | 0.02 | 0.04 | 0.4 | 0.4 | 0.04 | 0.04 | 539 | 402 | 3 | 4 | 5.9 | 7.4 |  |
|  | Min | 3 | 5 | 0.6 | 0.5 | 3.2 | 2.1 | 0.1 | 0.2 | 6.6 | 6.7 | 0.1 | 0.1 | 0.01 | 0.01 | 1.1 | 0.9 | 0.19 | 0.19 | 178 | 40 | 7 | 5 | 1.2 | 2.5 |  |
|  | Max | 26 | 43 | 7.6 | 16.4 | 24.5 | 26.8 | 0.6 | 11.4 | 8.1 | 7.8 | 0.3 | 0.5 | 0.06 | 0.11 | 2.5 | 2.4 | 0.28 | 0.29 | 1778 | 1215 | 18 | 17 | 14.1 | 18.5 |  |
|  | Kurt | 0 | -1 | 0.2 | 5 | -1.6 | -1.6 | 1.4 | 6.7 | 0.9 | -0.9 | -1.4 | -0.1 | -1.41 | -1.13 | 0.9 | 0.3 | -1.64 | -1.92 | -1 | 0 | 0 | 0 | -2.8 | -2.7 |  |
|  | Skew | 1 | 0 | 0.9 | 2 | 0.2 | 0.3 | 0.7 | 2.4 | -0.6 | -0.3 | 0.3 | 0.7 | 0.19 | 0.62 | -1.2 | 0.1 | -0.78 | -0.33 | 0 | 1 | 0 | -1 | -0.6 | 0.3 |  |
| 2006 | Avg. | 8 | 20 | 3.8 | 6 | 13.6 | 13.7 | 0.3 | 2.4 | 7.9 | 7.4 | 0.2 | 0.2 | 0.03 | 0.03 | 1.9 | 1.5 | 0.29 | 0.3 | 1003 | 789 | 11 | 10 | - | - |  |
|  | SD | 4 | 13 | 1.6 | 2.4 | 6.7 | 8 | 0.1 | 2 | 0.3 | 0.2 | 0.1 | 0.1 | 0.01 | 0.01 | 0.4 | 0.5 | 0.01 | 0.04 | 656 | 947 | 2 | 4 | - | - |  |
|  | Min | 3 | 4 | 1.2 | 1.8 | 4.4 | 2.9 | 0.2 | 0.2 | 7.6 | 7.2 | 0.1 | 0.1 | 0.01 | 0.01 | 1.4 | 0.8 | 0.27 | 0.27 | 288 | 25 | 8 | 5 | - | - |  |
|  | Max | 14 | 44 | 6.1 | 9.9 | 23.5 | 25.1 | 0.4 | 7.3 | 8.4 | 8 | 0.3 | 0.3 | 0.04 | 0.06 | 2.7 | 2.4 | 0.3 | 0.42 | 2175 | 3133 | 15 | 16 | - | - |  |
|  | Kurt | -2 | -1 | -0.8 | -0.3 | -1.5 | -1.4 | -0.7 | 2.7 | 1.4 | 4.6 | -1 | -0.9 | -1.37 | -0.2 | 0.3 | -0.1 | -0.91 | 9.76 | -1 | 2 | -1 | -1 | - | - |  |
|  | Skew | 0 | 1 | -0.1 | 0 | 0.1 | 0.1 | 0.3 | 1.3 | 1.6 | 1.9 | -0.2 | -0.4 | 0.03 | 0.61 | 0.5 | 0.4 | -1.05 | 2.98 | 1 | 2 | 0 | 0 | - | - |  |
| 2007 | Avg. | 8 | 21 | 3.9 | 4.1 | 13.7 | 13.8 | 0.4 | 6.8 | 7.9 | 7.6 | 0.2 | 0.2 | 0.02 | 0.05 | 2.2 | 1.6 | 0.15 | 0.24 | 916 | 768 | 11 | 9 | 8.2 | 14.2 |  |
|  | SD | 3 | 6 | 1.9 | 1.8 | 7.3 | 8.9 | 0.1 | 4.1 | 0.2 | 0.2 | 0 | 0.1 | 0.01 | 0.04 | 0.4 | 0.3 | 0.09 | 0.07 | 362 | 986 | 3 | 3 | 4.6 | 16.2 |  |
|  | Min | 4 | 10 | 1.6 | 1.3 | 3.4 | 1.4 | 0.2 | 2.6 | 7.6 | 7.4 | 0.1 | 0.1 | 0.01 | 0.02 | 1.5 | 1 | 0.06 | 0.1 | 340 | 57 | 8 | 5 | 2.7 | 3 |  |
|  | Max | 13 | 32 | 6.4 | 6.6 | 23.4 | 24.7 | 0.7 | 14.2 | 8.2 | 7.9 | 0.2 | 0.3 | 0.04 | 0.14 | 2.9 | 2.2 | 0.3 | 0.34 | 1540 | 3180 | 15 | 14 | 17.4 | 55.7 |  |
|  | Kurt | -1 | 0 | -1.7 | -1.3 | -1.7 | -1.9 | 3.9 | -0.4 | -0.6 | 0.4 | -0.3 | -0.9 | -0.31 | 1.38 | 0.3 | -0.5 | -1.06 | -0.42 | 0 | 3 | -2 | -2 | -0.3 | 3.5 |  |
|  | Skew | 1 | 0 | 0.4 | -0.1 | -0.2 | -0.1 | 1.7 | 0.9 | 0.2 | 0.5 | -0.1 | -0.4 | 0.37 | 1.41 | 0.2 | -0.3 | 0.54 | -0.39 | 0 | 2 | 0 | 0 | 0.5 | 2 |  |
| 2008 | Avg. | 11 | 17 | 4.9 | 5.7 | 13.3 | 13.3 | 0.3 | 3.3 | 7.9 | 7.6 | 0.2 | 0.2 | 0.03 | 0.04 | 1.8 | 1.6 | 0.14 | 0.22 | 670 | 803 | 11 | 9 | 6.3 | 8 |  |
|  | SD | 4 | 8 | 2 | 2.1 | 7.3 | 9.1 | 0.1 | 2.9 | 0.2 | 0.3 | 0.1 | 0.1 | 0.01 | 0.02 | 0.4 | 0.6 | 0.06 | 0.1 | 260 | 1062 | 2 | 4 | 3.4 | 6.2 |  |
|  | Min | 5 | 6 | 2.2 | 3.5 | 4.8 | 2.2 | 0.2 | 0.2 | 7.7 | 7.4 | 0 | 0.1 | 0.01 | 0.01 | 0.6 | 0.4 | 0.07 | 0.04 | 160 | 49 | 8 | 4 | 2 | 2.6 |  |
|  | Max | 19 | 28 | 9.1 | 11.3 | 23.8 | 25.8 | 0.5 | 8.1 | 8.3 | 8.2 | 0.2 | 0.4 | 0.04 | 0.07 | 2.5 | 2.2 | 0.26 | 0.38 | 1100 | 3610 | 13 | 13 | 11.6 | 24.2 |  |
|  | Kurt | 0 | -2 | 0.7 | 5 | -1.8 | -1.8 | 0.3 | -0.9 | 2.7 | 5.6 | -1.3 | -0.7 | -0.66 | -1.36 | 4 | 0.8 | 0.71 | 0.44 | 1 | 5 | -1 | -2 | -0.9 | 4.3 |  |
|  | Skew | 1 | 0 | 0.7 | 2 | 0.2 | 0.2 | 0 | 0.7 | 1.5 | 2.2 | -0.4 | 0.2 | -0.67 | 0.4 | -1.4 | -1.1 | 0.93 | 0 | 0 | 2 | 0 | 0 | 0.7 | 2 |  |
| 2009 | Avg. | 11 | 21 | 5 | 5.4 | 13 | 12.8 | 0.4 | 3.1 | 7.7 | 7.4 | 0.1 | 0.2 | 0.03 | 0.04 | 2.1 | 1.7 | 0.07 | 0.18 | 2537 | 825 | 10 | 10 | 4.6 | 5.8 |  |
|  | SD | 6 | 10 | 0.9 | 1 | 8.2 | 9.6 | 0.2 | 4.1 | 0.2 | 0.2 | 0 | 0 | 0.01 | 0.01 | 0.4 | 0.6 | 0.06 | 0.13 | 975 | 1131 | 2 | 4 | 2.2 | 6 |  |
|  | Min | 3 | 4 | 3.7 | 3.9 | 2.1 | 0.7 | 0.3 | 0.3 | 7.3 | 7.2 | 0.1 | 0.1 | 0.01 | 0.01 | 1.5 | 1 | 0.02 | 0.05 | 796 | 93 | 8 | 6 | 1.4 | 2.6 |  |
|  | Max | 28 | 43 | 6.5 | 7.1 | 23.1 | 25 | 0.8 | 13.8 | 7.9 | 7.8 | 0.2 | 0.2 | 0.04 | 0.06 | 2.9 | 2.7 | 0.22 | 0.52 | 4000 | 3900 | 14 | 15 | 8.3 | 24.6 |  |
|  | Kurt | 4 | 2 | -1 | -0.7 | -1.9 | -1.9 | 1 | 4.8 | 1.9 | 0 | 1.4 | 0.5 | -0.8 | -0.2 | 0.4 | -1.2 | 2.48 | 3.8 | 0 | 6 | 2 | -2 | -0.1 | 11.4 |  |
|  | Skew | 2 | 0 | 0.3 | 0.3 | -0.3 | -0.2 | 1.5 | 2.1 | -1.3 | 1.1 | -0.4 | -0.4 | 0.3 | 0.21 | 0.6 | 0.1 | 1.67 | 1.69 | -1 | 2 | 2 | 0 | 0.3 | 3.3 |  |
| 2010 | Avg. | 15 | 29 | 5.1 | 6.8 | 13.5 | 14.2 | 0.4 | 6.3 | 7.7 | 7.4 | 0.1 | 0.2 | 0.02 | 0.04 | 1.6 | 1.3 | 0.1 | 0.19 | 2097 | 644 | 10 | 9 | 10.7 | 7.6 |  |
|  | SD | 9 | 19 | 1.1 | 5.5 | 8 | 9.2 | 0.1 | 4.6 | 0.2 | 0.1 | 0.1 | 0.1 | 0.01 | 0.02 | 0.5 | 0.4 | 0.06 | 0.09 | 1243 | 540 | 2 | 4 | 8.7 | 3.3 |  |
|  | Min | 5 | 11 | 3.6 | 4.2 | 2.4 | 0 | 0.2 | 0.3 | 7.2 | 7.2 | 0.1 | 0.1 | 0.01 | 0.01 | 0.3 | 0.7 | 0.03 | 0.08 | 295 | 12 | 7 | 4 | 1 | 1.8 |  |
|  | Max | 31 | 77 | 7.7 | 24.1 | 24.1 | 26.2 | 0.5 | 14.3 | 8 | 7.6 | 0.2 | 0.3 | 0.04 | 0.07 | 2.2 | 2.4 | 0.25 | 0.39 | 4300 | 1582 | 14 | 15 | 31.8 | 14.1 |  |
|  | Kurt | -1 | 3 | 2.1 | 11.3 | -1.4 | -1.3 | 0.1 | -0.8 | 0.1 | 0 | -1 | -1.3 | -1.17 | -1.1 | 6.6 | 4.5 | 1.31 | 0.43 | 0 | -1 | -1 | -2 | 2 | 0.4 |  |
|  | Skew | 0 | 2 | 1.1 | 3.3 | -0.3 | -0.3 | -0.8 | 0.3 | -0.4 | 0.8 | -0.4 | 0 | 0.32 | -0.09 | -2.2 | 1.7 | 1.09 | 0.88 | 1 | 1 | 0 | 0 | 1.2 | 0.2 |  |
| 2011 | Avg. | 11 | 25 | 4.6 | 5 | 13.7 | 13.9 | 0.4 | 5.5 | 7.6 | 7.3 | 0.1 | 0.1 | 0.02 | 0.03 | 1.8 | 1.3 | 0.13 | 0.19 | 3360 | 1062 | 11 | 11 | 5.4 | 7.9 |  |
|  | SD | 6 | 9 | 1.4 | 0.9 | 7.2 | 8.4 | 0.2 | 3.7 | 0.2 | 0.2 | 0 | 0 | 0.01 | 0.01 | 0.4 | 0.7 | 0.05 | 0.09 | 945 | 1748 | 2 | 4 | 3 | 5.4 |  |
|  | Min | 3 | 13 | 3 | 3.8 | 2.9 | 2.1 | 0.1 | 1.2 | 7.2 | 6.9 | 0.1 | 0.1 | 0.01 | 0.02 | 1.3 | 0.6 | 0.05 | 0.1 | 1445 | 179 | 8 | 6 | 2.1 | 1.9 |  |
|  | Max | 20 | 42 | 7.8 | 7.4 | 24 | 26.4 | 1 | 12.9 | 8.1 | 7.7 | 0.2 | 0.2 | 0.03 | 0.04 | 2.6 | 2.9 | 0.26 | 0.32 | 4850 | 6200 | 15 | 18 | 11.5 | 17.8 |  |
|  | Kurt | -1 | 0 | 1.4 | 4.1 | -1.2 | -1.3 | 3.9 | -0.2 | 0.8 | -0.5 | 0.8 | 1.9 | -0.42 | -1.01 | -0.6 | 2.1 | 2.01 | -1.58 | 1 | 10 | -1 | 0 | -0.4 | -1 |  |
|  | Skew | 0 | 1 | 1.2 | 1.6 | -0.1 | 0.1 | 1.7 | 0.8 | 0.6 | -0.1 | 0.8 | 1.6 | -0.54 | -0.23 | 0.7 | 1.5 | 0.99 | 0.56 | 0 | 3 | 1 | 1 | 0.7 | 0.4 |  |
| 2012 | Avg. | 14 | 35 | 3.4 | 3.5 | 14.4 | 14.8 | 0.4 | 14.2 | 7.9 | 7.6 | 0.1 | 0.2 | 0.03 | 0.06 | 2 | 1.2 | 0.12 | 0.28 | 1001 | 690 | 11 | 8 | 8 | 7 |  |
|  | SD | 7 | 10 | 0.9 | 0.7 | 5.7 | 7.9 | 0.1 | 4.1 | 0.3 | 0.2 | 0 | 0 | 0.01 | 0.03 | 0.3 | 0.2 | 0.08 | 0.17 | 374 | 1428 | 3 | 3 | 4.9 | 4.7 |  |
|  | Min | 5 | 22 | 1.7 | 2.7 | 7.8 | 5.5 | 0.3 | 8.7 | 7.5 | 7.2 | 0.1 | 0.1 | 0.02 | 0.03 | 1.5 | 1 | 0.04 | 0.13 | 480 | 11 | 6 | 5 | 1.8 | 1.1 |  |
|  | Max | 27 | 53 | 5.3 | 5.3 | 24 | 25.7 | 0.6 | 20.8 | 8.6 | 7.9 | 0.2 | 0.2 | 0.04 | 0.12 | 2.4 | 1.5 | 0.35 | 0.72 | 1730 | 4775 | 18 | 13 | 15.7 | 16.8 |  |
|  | Kurt | 0 | 0 | 1 | 2.5 | -1.1 | -1.7 | 0.2 | -1 | 0.8 | -1.1 | -0.8 | -1.2 | -1.18 | -0.16 | 0 | -1.6 | 7.56 | 4.19 | 1 | 8 | 0 | -1 | -1.1 | 0.4 |  |
|  | Skew | 0 | 1 | 0.1 | 1.2 | 0.6 | 0.1 | 0.7 | 0.3 | 1 | -0.4 | -0.2 | -0.1 | -0.28 | 0.75 | -0.3 | 0.2 | 2.51 | 1.86 | 1 | 3 | 1 | 0 | 0.4 | 0.6 |  |
| 2013 | Avg. | 12 | 28 | 3.9 | 4.3 | 12.9 | 13.9 | 0.4 | 9.6 | 7.6 | 7.7 | 0.1 | 0.2 | 0.02 | 0.04 | 1.8 | 1.3 | 0.11 | 0.23 | 3044 | 310 | 12 | 11 | 5.2 | 5.7 |  |
|  | SD | 7 | 5 | 2.1 | 1.1 | 7.3 | 8.3 | 0.1 | 4.7 | 0.8 | 0.3 | 0 | 0 | 0.01 | 0.02 | 0.4 | 0.3 | 0.07 | 0.08 | 2061 | 333 | 2 | 4 | 3.7 | 3.3 |  |
|  | Min | 2 | 23 | 0.3 | 2.6 | 3.7 | 2.8 | 0.2 | 2.8 | 5.9 | 7.3 | 0.1 | 0.1 | 0.01 | 0.02 | 1 | 1 | 0.02 | 0.11 | 840 | 26 | 9 | 5 | 2.1 | 2.1 |  |
|  | Max | 25 | 38 | 7.8 | 6.2 | 23.8 | 25.9 | 0.5 | 17.6 | 8.3 | 8.4 | 0.2 | 0.2 | 0.04 | 0.08 | 2.4 | 2 | 0.27 | 0.43 | 6600 | 1210 | 16 | 20 | 12.7 | 14 |  |
|  | Kurt | -1 | 1 | -0.3 | -0.8 | -1.7 | -1.7 | 2 | -1 | 1.5 | 0.9 | 0.4 | 0.1 | -1.1 | 1.24 | -0.5 | 2.9 | 0.58 | 3.02 | -1 | 6 | 0 | 1 | 1 | 2.6 |  |
|  | Skew | 0 | 1 | 0.1 | 0.2 | 0 | 0 | -0.4 | 0.3 | -1.5 | 1 | 0 | -1.1 | -0.03 | 1.19 | -0.4 | 1.4 | 1.02 | 1.18 | 1 | 2 | 0 | 1 | 1.5 | 1.4 |  |
| 2014 | Avg. | 14 | 26 | 4 | 4.1 | 12.5 | 12.5 | 0.5 | 7.7 | 7 | 7.1 | 0.1 | 0.2 | 0.03 | 0.04 | 1.9 | 1.4 | 0.14 | 0.18 | 2180 | 1191 | 12 | 10 | 6.1 | 8 |  |
|  | SD | 9 | 9 | 1.6 | 1.2 | 8.3 | 9 | 0.2 | 3.8 | 0.7 | 0.4 | 0 | 0.1 | 0.01 | 0.02 | 0.4 | 0.6 | 0.09 | 0.09 | 1283 | 1592 | 3 | 4 | 4.1 | 7.8 |  |
|  | Min | 4 | 12 | 1.5 | 2.1 | 0.2 | 0.4 | 0.3 | 1.4 | 5.7 | 6.1 | 0.1 | 0.1 | 0.02 | 0.02 | 1 | 0.7 | 0.05 | 0.07 | 620 | 214 | 8 | 5 | 1.8 | 1.5 |  |
|  | Max | 36 | 38 | 7 | 5.5 | 23.3 | 24 | 1 | 14.9 | 7.7 | 7.5 | 0.2 | 0.4 | 0.04 | 0.09 | 2.3 | 2.6 | 0.29 | 0.41 | 4100 | 4519 | 16 | 15 | 13.7 | 23.8 |  |
|  | Kurt | 2 | -1 | -0.9 | -1.5 | -1.7 | -1.8 | 2.8 | 0 | -0.7 | 4.2 | -0.2 | 3.8 | -0.2 | 1.98 | 3.1 | 1.2 | -1.07 | 1.57 | -1 | 2 | -2 | -2 | -0.9 | 0 |  |
|  | Skew | 1 | 0 | 0.2 | -0.4 | -0.1 | 0 | 1.6 | 0.1 | -0.8 | -1.9 | 0 | 1.5 | 0.19 | 1.09 | -1.3 | 1.3 | 0.58 | 1.12 | 0 | 2 | 0 | 0 | 0.7 | 1.1 |  |
| 2015 | Avg. | 13 | 30 | 4.9 | 4.6 | 13.4 | 13.8 | 0.7 | 13.1 | 7.7 | 7.4 | 0.1 | 0.2 | 0.04 | 0.05 | 1.9 | 1.2 | - | - | 2915 | 711 | 11 | 9 | 5.5 | 2.9 |  |
|  | SD | 6 | 6 | 1.9 | 1.2 | 7.9 | 9.2 | 0.3 | 3.9 | 0.2 | 0.2 | 0 | 0.1 | 0.01 | 0.01 | 0.4 | 0.4 | - | - | 1839 | 453 | 3 | 3 | 6.4 | 2.1 |  |
|  | Min | 7 | 20 | 2.6 | 3.2 | 2.9 | 0.9 | 0.4 | 5.2 | 7.3 | 7 | 0.1 | 0.1 | 0.02 | 0.03 | 1.1 | 0.6 | - | - | 885 | 20 | 8 | 5 | 1.1 | 0.9 |  |
|  | Max | 26 | 40 | 7.9 | 6.2 | 23.5 | 25.2 | 1.3 | 18.6 | 8 | 7.8 | 0.2 | 0.3 | 0.06 | 0.09 | 2.6 | 2.3 | - | - | 6000 | 1814 | 16 | 13 | 20.1 | 6.7 |  |
|  | Kurt | 1 | -1 | -1.4 | -1.6 | -1.7 | -1.5 | 0.6 | 0.1 | 0.4 | -0.1 | 0.1 | -0.6 | 1.51 | 7.23 | 0.3 | 2.3 | - | - | -1 | 3 | -2 | -2 | 2.1 | -0.4 |  |
|  | Skew | 1 | 0 | 0.3 | 0.4 | 0 | -0.1 | 1.1 | -0.3 | -0.3 | 0 | -0.9 | -0.2 | 1.32 | 2.3 | -0.3 | 1.1 | - | - | 1 | 1 | 0 | 0 | 1.8 | 0.9 |  |
| 2016 | Avg. | 19 | 31 | 4.1 | 4 | 13.7 | 14.7 | 1 | 11.5 | 7.4 | 7.3 | 0.1 | 0.2 | 0.03 | 0.06 | 2 | 1.3 | 0.14 | 0.3 | 2038 | 661 | 11 | 9 | 6.5 | 6.6 |  |
|  | SD | 9 | 7 | 0.8 | 0.8 | 7.6 | 8.5 | 1 | 4.7 | 0.2 | 0.3 | 0 | 0.1 | 0.02 | 0.03 | 0.4 | 0.3 | 0.03 | 0.08 | 937 | 668 | 3 | 4 | 4.6 | 8.1 |  |
|  | Min | 8 | 20 | 2.9 | 2.9 | 2.9 | 3.9 | 0.3 | 3.7 | 7.1 | 6.9 | 0.1 | 0.1 | 0.02 | 0.02 | 1 | 0.7 | 0.09 | 0.2 | 935 | 128 | 8 | 5 | 1 | 0.8 |  |
|  | Max | 36 | 43 | 5.6 | 5.2 | 23.6 | 26.2 | 3 | 18.5 | 7.8 | 7.7 | 0.2 | 0.2 | 0.06 | 0.12 | 2.7 | 1.8 | 0.21 | 0.43 | 3708 | 2449 | 18 | 17 | 12.5 | 29.9 |  |
|  | Kurt | 1 | 0 | -0.3 | -1 | -1.4 | -1.7 | 1.6 | -0.7 | 0.1 | -0.2 | -0.6 | 0.4 | -0.93 | 0.1 | 1.1 | 0.2 | -0.36 | -1.08 | -1 | 4 | 1 | -1 | -2.1 | 7.2 |  |
|  | Skew | 1 | 0 | 0.3 | -0.1 | 0 | 0.1 | 1.7 | 0.4 | -0.2 | -0.2 | -0.7 | -0.7 | 0.53 | 0.97 | -0.6 | -0.2 | 0.58 | 0.57 | 0 | 2 | 1 | 1 | 0 | 2.5 |  |
| 2017 | Avg. | 15 | 25 | 4.2 | 4.4 | 13.9 | 14.3 | 0.5 | 8.4 | 8.1 | 7.7 | 0.1 | 0.2 | 0.03 | 0.06 | 2 | 1.4 | 0.23 | 0.36 | 2042 | 1175 | 11 | 9 | 11.2 | 8.3 |  |
|  | SD | 9 | 7 | 1.1 | 0.8 | 6.4 | 8.3 | 0.2 | 4.7 | 0.4 | 0.3 | 0 | 0 | 0.01 | 0.02 | 0.5 | 0.3 | 0.08 | 0.09 | 672 | 1267 | 2 | 3 | 7.7 | 6.8 |  |
|  | Min | 6 | 10 | 2.5 | 2.8 | 4.6 | 2.9 | 0.4 | 0.6 | 7.6 | 7.3 | 0.1 | 0.1 | 0.01 | 0.03 | 1.4 | 1 | 0.11 | 0.24 | 1107 | 13 | 8 | 6 | 3.1 | 1.7 |  |
|  | Max | 38 | 38 | 6.1 | 5.7 | 22.5 | 25.3 | 1 | 15.6 | 8.7 | 8.3 | 0.2 | 0.2 | 0.06 | 0.08 | 2.8 | 1.8 | 0.38 | 0.56 | 2864 | 4200 | 14 | 13 | 24.6 | 26.2 |  |
|  | Kurt | 5 | 1 | -0.6 | -0.4 | -1.3 | -1.7 | 10.1 | -0.5 | -1.2 | -0.3 | 0.8 | 0.4 | -0.05 | -1.59 | -1.4 | -0.4 | 0.56 | 1.83 | -2 | 3 | 0 | -2 | -1.1 | 4.2 |  |
|  | Skew | 2 | 0 | -0.1 | -0.3 | -0.2 | -0.2 | 3.1 | -0.4 | 0.4 | 0.6 | -0.2 | -1.1 | 0.7 | 0.38 | 0.2 | 0 | 0.48 | 1.19 | 0 | 2 | 0 | 0 | 0.7 | 2 |  |
| 2018 | Avg. | 13 | 20 | 4.2 | 4.2 | 13 | 13.2 | 0.4 | 6.8 | 7.7 | 7.5 | 0.1 | 0.1 | 0.02 | 0.03 | 1.8 | 1.1 | 0.16 | 0.28 | 3146 | 1646 | 12 | 10 | 7.5 | 7.1 |  |
|  | SD | 7 | 7 | 1.2 | 1 | 7.9 | 9.3 | 0.1 | 4.6 | 0.2 | 0.3 | 0 | 0.1 | 0.01 | 0.01 | 0.4 | 0.4 | 0.01 | 0.1 | 930 | 1102 | 3 | 4 | 4.4 | 5.8 |  |
|  | Min | 4 | 12 | 2.2 | 3.3 | 1.5 | 1.1 | 0.2 | 2.7 | 7.4 | 7.2 | 0.1 | 0 | 0.01 | 0.01 | 1.4 | 0.7 | 0.15 | 0.15 | 1553 | 230 | 8 | 5 | 1.7 | 1.4 |  |
|  | Max | 24 | 32 | 6.5 | 6.9 | 23.5 | 25.4 | 0.6 | 19.3 | 7.9 | 7.9 | 0.2 | 0.2 | 0.04 | 0.06 | 2.6 | 1.9 | 0.2 | 0.52 | 4550 | 3580 | 18 | 16 | 15.6 | 22 |  |
|  | Kurt | -1 | -1 | 0.4 | 5.5 | -1.6 | -1.9 | 0.7 | 4.6 | -1.7 | -1.4 | -0.5 | -0.8 | -1.03 | -0.65 | 1.3 | 2 | 5.15 | 2.22 | 0 | -1 | 2 | -2 | -0.6 | 3.3 |  |
|  | Skew | 0 | 1 | 0.4 | 2.1 | 0 | 0.1 | 0.4 | 1.9 | 0.1 | 0.4 | 0.3 | 0.2 | 0.45 | 0.36 | 1.3 | 1.3 | 2.18 | 1.24 | 0 | 0 | 1 | 0 | 0.4 | 1.6 |  |
| 2019 | Avg. | 10 | 16 | 3.5 | 4 | 13 | 13.6 | 0.5 | 6.4 | 7.8 | 7.2 | 0.1 | 0.1 | 0.02 | 0.04 | 2.2 | 1.1 | - | - | 3499 | 1027 | 12 | 10 | 4 | 4.8 |  |
|  | SD | 7 | 6 | 1.1 | 1.2 | 7.6 | 9 | 0.2 | 4 | 0.2 | 0.2 | 0 | 0 | 0 | 0.02 | 0.6 | 0.2 | - | - | 1162 | 1129 | 3 | 4 | 2.5 | 3.4 |  |
|  | Min | 4 | 1 | 1.9 | 2.6 | 4.8 | 2.5 | 0.3 | 0.2 | 7.4 | 6.9 | 0 | 0 | 0.02 | 0.01 | 1.6 | 0.8 | - | - | 1750 | 46 | 8 | 6 | 0.9 | 1.3 |  |
|  | Max | 21 | 26 | 5.2 | 6.6 | 23.5 | 25.8 | 0.9 | 12.5 | 8.1 | 7.5 | 0.2 | 0.2 | 0.03 | 0.08 | 3.2 | 1.6 | - | - | 5020 | 3367 | 17 | 16 | 9.6 | 12.7 |  |
|  | Kurt | -1 | 2 | -1.1 | 0 | -2 | -1.9 | 2 | -0.8 | -1.3 | -1.3 | 3.4 | -1.8 | -0.77 | 1.72 | -1 | 0.8 | - | - | -1 | 1 | -1 | -2 | 1 | 1.9 |  |
|  | Skew | 1 | -1 | 0.2 | 0.7 | 0.2 | 0.1 | 1.5 | 0.3 | 0 | -0.1 | -1.8 | -0.2 | 0.06 | 1.22 | 0.7 | 1 | - | - | 0 | 2 | 0 | 0 | 0.9 | 1.5 |  |

Note: a and b represent the ULPR and DLPR study sites respectively

| Table S2, Aquatic life standards used for WQI computation | | |
| --- | --- | --- |
| Parameter | Aquatic Life Criteria | References |
| NH_3_-N (mg L^-1^) | 0.203 | (Haigler, 2002) |
| Chlorophyll-a (µg L^-1^) | 20 | (NJDEP, 2020) |
| DO (mg L^-1^) | 6.5 | (CCME (Canadian Council of Ministers of the Environment), 1999) |
| F. Coli (cfu 100mL^-1^) | 200 | (Wade et al., 2003) |
| Nitrate (mg L^-1^) | 3 | (Nordin & Pommen, 2009) |
| Nitrite (mg L^-1^) | 0.06 | (PHILMINAQ, 2010) |
| DOC (mg L^-1^) | 8.3 | (Leusch et al., 2012) |
| Ortho-P (mg L^-1^) | 0.1 | (PHILMINAQ, 2010) |
| pH | 8 | (Prabu et al., 2008) |
| TSS (mg L^-1^) | 25 | (Government of Alberta, 2014) |
| Temperature (°C) | 25 - 35 | (Olubanjo & Adeleke, 2020) |
| Salinity (ppth) | 0.5 | (USEPA, 2006) |

| Table S3 Monthly forecast values for the upper and Lower Passaic River study areas | | | | | | | | | | | | | |
| --- | --- | --- | --- | --- | --- | --- | --- | --- | --- | --- | --- | --- | --- |
|  | | ULPR | | | | | | LLPR | | | | | |
| Month/Year | | Point.Forecast | | Lo.80 | Hi.80 | Lo.95 | Hi.95 | Point.Forecast | | Lo.80 | Hi.80 | Lo.95 | Hi.95 |
| Jan-20 | | 26.2 | | 7.5 | 45.0 | -2.5 | 54.9 | 38.4 | | 8.8 | 67.9 | -6.8 | 83.5 |
| Feb-20 | | 27.0 | | 7.1 | 46.9 | -3.5 | 57.5 | 47.7 | | 13.9 | 81.5 | -4.1 | 99.4 |
| Mar-20 | | 22.2 | | 2.0 | 42.4 | -8.7 | 53.1 | 35.9 | | 0.8 | 71.0 | -17.8 | 89.6 |
| Apr-20 | | 23.0 | | 2.5 | 43.5 | -8.3 | 54.4 | 42.3 | | 6.8 | 77.8 | -12.0 | 96.6 |
| May-20 | | 35.1 | | 14.4 | 55.9 | 3.4 | 66.9 | 60.5 | | 24.8 | 96.2 | 6.0 | 115.0 |
| Jun-20 | | 42.3 | | 21.3 | 63.4 | 10.1 | 74.5 | 75.8 | | 40.0 | 111.5 | 21.1 | 130.4 |
| Jul-20 | | 39.3 | | 18.0 | 60.6 | 6.7 | 71.9 | 72.2 | | 36.4 | 107.9 | 17.5 | 126.8 |
| Aug-20 | | 34.8 | | 13.2 | 56.3 | 1.8 | 67.8 | 73.3 | | 37.6 | 109.1 | 18.6 | 128.0 |
| Sep-20 | | 34.7 | | 12.9 | 56.5 | 1.3 | 68.1 | 94.2 | | 58.4 | 130.0 | 39.5 | 148.9 |
| Oct-20 | | 34.9 | | 12.8 | 57.0 | 1.1 | 68.7 | 99.6 | | 63.9 | 135.4 | 45.0 | 154.3 |
| Nov-20 | | 35.1 | | 12.7 | 57.5 | 0.9 | 69.3 | 76.7 | | 40.9 | 112.4 | 22.0 | 131.4 |
| Dec-20 | | 30.0 | | 7.4 | 52.6 | -4.6 | 64.6 | 67.9 | | 32.2 | 103.7 | 13.2 | 122.6 |
| Jan-21 | | 26.0 | | 2.6 | 49.4 | -9.7 | 61.8 | 47.2 | | 11.0 | 83.4 | -8.2 | 102.6 |
| Feb-21 | | 25.0 | | 1.2 | 48.7 | -11.4 | 61.3 | 52.3 | | 15.9 | 88.6 | -3.4 | 107.9 |
| Mar-21 | | 20.1 | | -4.0 | 44.2 | -16.7 | 56.9 | 38.2 | | 1.8 | 74.6 | -17.5 | 93.9 |
| Apr-21 | | 21.0 | | -3.4 | 45.4 | -16.3 | 58.3 | 43.3 | | 6.9 | 79.8 | -12.4 | 99.1 |
| May-21 | | 33.1 | | 8.4 | 57.8 | -4.7 | 70.8 | 60.8 | | 24.4 | 97.3 | 5.0 | 116.6 |
| Jun-21 | | 40.3 | | 15.3 | 65.2 | 2.1 | 78.5 | 75.7 | | 39.2 | 112.2 | 19.9 | 131.5 |
| Jul-21 | | 37.3 | | 12.0 | 62.5 | -1.4 | 75.9 | 71.9 | | 35.4 | 108.4 | 16.1 | 127.7 |
| Aug-21 | | 32.7 | | 7.1 | 58.3 | -6.4 | 71.8 | 72.9 | | 36.4 | 109.4 | 17.1 | 128.7 |
| Sep-21 | | 32.6 | | 6.8 | 58.5 | -6.9 | 72.2 | 93.8 | | 57.3 | 130.2 | 38.0 | 149.6 |
| Oct-21 | | 32.9 | | 6.7 | 59.0 | -7.1 | 72.8 | 99.2 | | 62.7 | 135.7 | 43.4 | 155.0 |
| Nov-21 | | 33.1 | | 6.6 | 59.5 | -7.3 | 73.5 | 76.2 | | 39.7 | 112.7 | 20.4 | 132.0 |
| Dec-21 | | 28.0 | | 1.3 | 54.6 | -12.9 | 68.8 | 67.4 | | 30.9 | 103.9 | 11.6 | 123.2 |
| Jan-22 | | 24.0 | | -3.5 | 51.4 | -18.0 | 65.9 | 46.7 | | 9.7 | 83.6 | -9.8 | 103.2 |
| Feb-22 | | 22.9 | | -4.9 | 50.8 | -19.7 | 65.5 | 51.8 | | 14.6 | 88.9 | -5.0 | 108.5 |
| Mar-22 | | 18.1 | | -10.1 | 46.3 | -25.0 | 61.2 | 37.7 | | 0.5 | 74.9 | -19.2 | 94.5 |
| Apr-22 | | 18.9 | | -9.6 | 47.4 | -24.7 | 62.5 | 42.8 | | 5.6 | 80.0 | -14.1 | 99.7 |
| May-22 | | 31.0 | | 2.2 | 59.9 | -13.1 | 75.1 | 60.3 | | 23.1 | 97.5 | 3.4 | 117.2 |
| Jun-22 | | 38.2 | | 9.1 | 67.4 | -6.4 | 82.8 | 75.2 | | 38.0 | 112.4 | 18.2 | 132.1 |
| Jul-22 | | 35.2 | | 5.8 | 64.7 | -9.8 | 80.3 | 71.4 | | 34.1 | 108.6 | 14.4 | 128.3 |
| Aug-22 | | 30.7 | | 0.9 | 60.4 | -14.9 | 76.2 | 72.4 | | 35.2 | 109.6 | 15.5 | 129.3 |
| Sep-22 | | 30.6 | | 0.5 | 60.7 | -15.4 | 76.6 | 93.2 | | 56.0 | 130.5 | 36.3 | 150.2 |
| Oct-22 | | 30.8 | | 0.4 | 61.2 | -15.7 | 77.3 | 98.7 | | 61.4 | 135.9 | 41.7 | 155.6 |
| Nov-22 | | 31.0 | | 0.3 | 61.7 | -15.9 | 77.9 | 75.7 | | 38.4 | 112.9 | 18.7 | 132.6 |
| Dec-22 | | 25.9 | | -5.1 | 56.9 | -21.5 | 73.3 | 66.9 | | 29.7 | 104.2 | 10.0 | 123.9 |
| Jan-23 | | 21.9 | | -9.8 | 53.6 | -26.6 | 70.4 | 46.1 | | 8.4 | 83.8 | -11.5 | 103.8 |
| Feb-23 | | 20.9 | | -11.3 | 53.0 | -28.3 | 70.0 | 51.2 | | 13.4 | 89.1 | -6.7 | 109.2 |
| Mar-23 | | 16.0 | | -16.5 | 48.5 | -33.7 | 65.7 | 37.2 | | -0.8 | 75.1 | -20.9 | 95.2 |
| Apr-23 | | 16.9 | | -16.0 | 49.7 | -33.4 | 67.1 | 42.3 | | 4.3 | 80.3 | -15.8 | 100.4 |
| May-23 | | 29.0 | | -4.2 | 62.2 | -21.8 | 79.8 | 59.8 | | 21.8 | 97.8 | 1.7 | 117.9 |
| Jun-23 | | 36.2 | | 2.6 | 69.7 | -15.1 | 87.4 | 74.7 | | 36.7 | 112.7 | 16.5 | 132.8 |
| Jul-23 | | 33.2 | | -0.7 | 67.0 | -18.6 | 85.0 | 70.9 | | 32.9 | 108.9 | 12.7 | 129.0 |
| Aug-23 | | 28.6 | | -5.6 | 62.8 | -23.7 | 80.9 | 71.9 | | 33.9 | 109.9 | 13.8 | 130.0 |
| Sep-23 | | 28.5 | | -6.0 | 63.1 | -24.3 | 81.3 | 92.7 | | 54.7 | 130.7 | 34.6 | 150.8 |
| Oct-23 | | 28.8 | | -6.1 | 63.6 | -24.5 | 82.1 | 98.1 | | 60.1 | 136.1 | 40.0 | 156.3 |
| Nov-23 | | 29.0 | | -6.2 | 64.1 | -24.8 | 82.7 | 75.1 | | 37.1 | 113.1 | 17.0 | 133.3 |
| Dec-23 | | 23.9 | | -11.6 | 59.3 | -30.4 | 78.1 | 66.4 | | 28.4 | 104.4 | 8.2 | 124.5 |
| Jan-24 | | 19.9 | | -16.4 | 56.1 | -35.6 | 75.3 | 45.6 | | 7.1 | 84.1 | -13.2 | 104.5 |
| Feb-24 | | 18.8 | | -17.9 | 55.5 | -37.3 | 74.9 | 50.7 | | 12.1 | 89.4 | -8.4 | 109.8 |
| Mar-24 | | 14.0 | | -23.1 | 51.0 | -42.7 | 70.6 | 36.6 | | -2.1 | 75.4 | -22.6 | 95.9 |
| Apr-24 | | 14.8 | | -22.6 | 52.2 | -42.4 | 72.0 | 41.8 | | 3.0 | 80.6 | -17.5 | 101.1 |
| May-24 | | 26.9 | | -10.8 | 64.7 | -30.8 | 84.7 | 59.3 | | 20.5 | 98.0 | -0.1 | 118.6 |
| Jun-24 | | 34.1 | | -4.0 | 72.2 | -24.2 | 92.4 | 74.1 | | 35.3 | 112.9 | 14.8 | 133.5 |
| Jul-24 | | 31.1 | | -7.4 | 69.6 | -27.7 | 90.0 | 70.3 | | 31.5 | 109.1 | 11.0 | 129.7 |
| Aug-24 | | 26.6 | | -12.3 | 65.4 | -32.8 | 85.9 | 71.4 | | 32.6 | 110.2 | 12.0 | 130.7 |
| Sep-24 | | 26.5 | | -12.7 | 65.7 | -33.4 | 86.4 | 92.2 | | 53.4 | 131.0 | 32.9 | 151.5 |
| Oct-24 | | 26.7 | | -12.8 | 66.2 | -33.7 | 87.1 | 97.6 | | 58.8 | 136.4 | 38.3 | 157.0 |
| Nov-24 | | 26.9 | | -12.9 | 66.8 | -34.0 | 87.9 | 74.6 | | 35.8 | 113.4 | 15.3 | 134.0 |
| Dec-24 | | 21.8 | | -18.4 | 62.0 | -39.7 | 83.3 | 65.9 | | 27.1 | 104.7 | 6.5 | 125.2 |
| Jan-25 | | 17.8 | | -23.1 | 58.7 | -44.8 | 80.4 | 45.1 | | 5.8 | 84.4 | -15.0 | 105.2 |
| Feb-25 | | 16.8 | | -24.6 | 58.1 | -46.5 | 80.1 | 50.2 | | 10.7 | 89.7 | -10.2 | 110.6 |
| Mar-25 | | 11.9 | | -29.8 | 53.7 | -52.0 | 75.8 | 36.1 | | -3.4 | 75.7 | -24.4 | 96.6 |
| Apr-25 | | 12.8 | | -29.4 | 54.9 | -51.7 | 77.2 | 41.3 | | 1.7 | 80.8 | -19.3 | 101.8 |
| May-25 | | 24.9 | | -17.6 | 67.4 | -40.2 | 89.9 | 58.7 | | 19.1 | 98.3 | -1.8 | 119.3 |
| Jun-25 | | 32.1 | | -10.8 | 75.0 | -33.5 | 97.7 | 73.6 | | 34.0 | 113.2 | 13.0 | 134.2 |
| Jul-25 | | 29.1 | | -14.2 | 72.3 | -37.1 | 95.2 | 69.8 | | 30.2 | 109.4 | 9.2 | 130.4 |
| Aug-25 | | 24.5 | | -19.1 | 68.1 | -42.2 | 91.2 | 70.9 | | 31.2 | 110.5 | 10.3 | 131.4 |
| Sep-25 | | 24.4 | | -19.6 | 68.4 | -42.9 | 91.7 | 91.7 | | 52.1 | 131.3 | 31.1 | 152.3 |
| Oct-25 | | 24.7 | | -19.7 | 69.0 | -43.2 | 92.5 | 97.1 | | 57.5 | 136.7 | 36.5 | 157.7 |
| Nov-25 | | 24.9 | | -19.9 | 69.6 | -43.5 | 93.3 | 74.1 | | 34.5 | 113.7 | 13.5 | 134.7 |
| Dec-25 | | 19.8 | | -25.3 | 64.8 | -49.2 | 88.7 | 65.4 | | 25.7 | 105.0 | 4.7 | 126.0 |

| Table S4, Correlation coefficients (R^2^) of physicochemical parameters between observed and predicted values by the PMF model | | | | | | | | |
| --- | --- | --- | --- | --- | --- | --- | --- | --- |
|  | Downstream LPR Monitoring Sites | | | | Upstream LPR Monitoring Sites | | | |
| Parameters | Intercept | Slope | SE | R^2^ | Intercept | Slope | SE | R^2^ |
| Fecal Coliform | 0.05616 | 0.99 | 0.155437 | 0.99 | 0.0941492 | 0.99 | 0.0679589 | 1.00 |
| Nitrate | 0.00001 | 0.99 | 0.000003 | 1.00 | 0.0000032 | 0.99 | 0.0000030 | 1.00 |
| Orthophosphate | 0.00002 | 0.99 | 0.000005 | 1.00 | 0.0000002 | 0.99 | 0.0000003 | 1.00 |
| TSS | 0.00003 | 1.00 | 0.000040 | 1.00 | -0.0000350 | 1.00 | 0.0099732 | 0.99 |


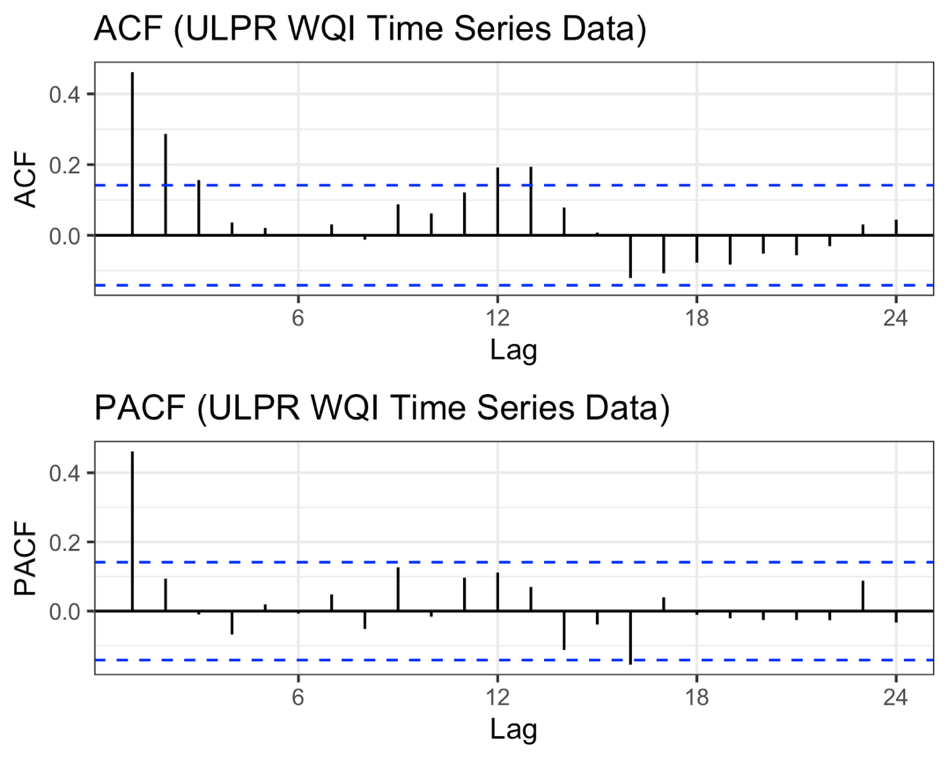

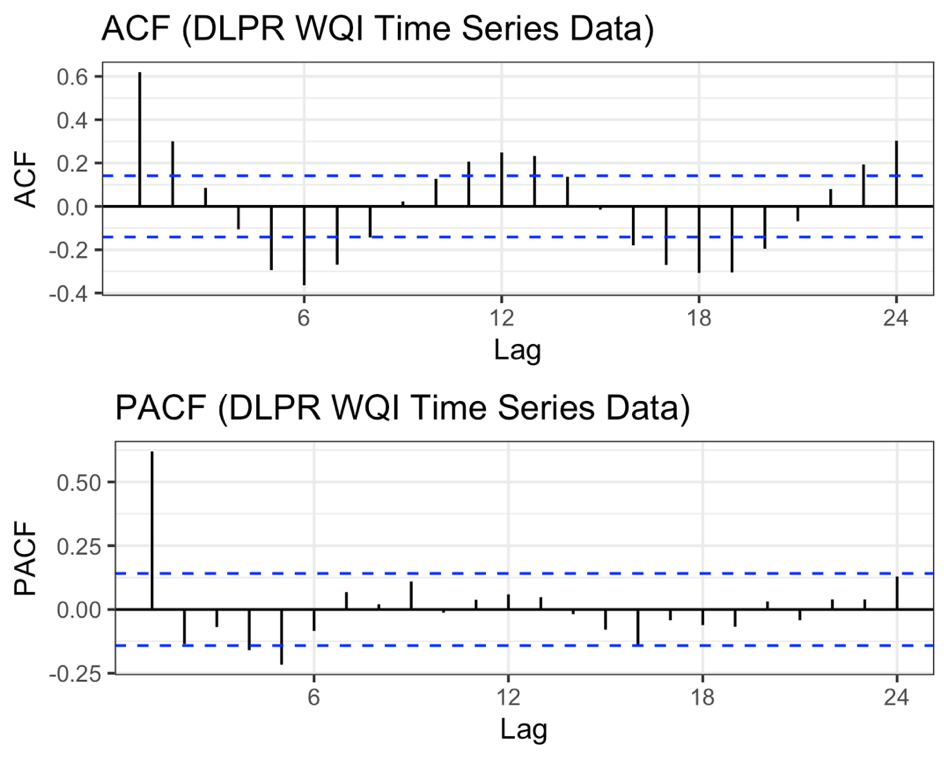


Figs. S1 and S2, ACF and PACF plots for ULPR and DLPR water quality data respectively, showing seasonality and non-stationarity of the data

**References**

CCME (Canadian Council of Ministers of the Environment). (1999). Canadian Water Quality Guidelines for the Protection of Aquatic Life - Dissolved Oxygen (Freshwater). In *Canadian environmental quality guidelines*.

Government of Alberta. (2014). *Environmental Quality Guidelines for Alberta Surface Waters*. http://aep.alberta.ca/water/education-guidelines/documents/EnvironmentalQualitySurfaceWaters-2014.pdf

Haigler, B. (2002). Final TMDL Development for Thacker Creek \Low Dissolved Oxygen / Organic Loading Ammonia as Nitrogen. In *Water Quality Branch, ADEM* (Issue February).

Leusch, F., Prochazka, E., Carswell, S., & Escher, B. (2012). Optimising Micropollutants Extraction for Analysis of Water Samples: Comparison of Different Solid Phase Materials and Liquid-Liquid Extraction. *Science Forum and Stakeholder Engagement: Building Linkages, Collaboration and Science Quality*, *June*, 191–195.

NJDEP. (2020). *Surface Water Quality Standards*.

Nordin, R. N., & Pommen, L. W. (2009). Water Quality Guidelines for Nitrogen (Nitrate, Nitrite, and Ammonia) - Overview Report Update. *Water Stewardship Division, Ministry of Environment, Province of British Columbia, Canada*, 29. https://www2.gov.bc.ca/assets/gov/environment/air-land-water/water/waterquality/wqgs-wqos/approved-wqgs/nitrogen-overview.pdf%0Ahttp://a100.gov.bc.ca/pub/eirs/finishDownloadDocument.do?subdocumentId=9021

Olubanjo, O. O., & Adeleke, E. B. (2020). Assessment of Physico-chemical Properties and Water Quality of River Osse, Kogi State. *Applied Research Journal of Environmental Engineering*, *3*(1), 21–30. https://doi.org/10.47721/arjee20200301030

PHILMINAQ. (2010). *Water Quality Criteria and Standards for Freshwater and Marine Aquaculture*.

Prabu, P. C., Teklemariam, Z., Nigusse, T., Rajeshkumar, S., Wondimu, L., Negassa, A., Debebe, E., Aga, E., Andargie, A., & Keneni, A. (2008). Characterisation of sewage wastewater and assessment of downstream pollution along Huluka River of Ambo, Ethiopia. *Maejo International Journal of Science and Technology*, *2*(2), 298–307.

USEPA. (2006). Voluntary Estuary Monitoring Manual Chapter 14: Salinity March 2006. *Volunteer Estuary Monitoring Manual: A Methods Manual*, *March*, 14.1-14.5.

Wade, T. J., Pai, N., Eisenberg, J. N. S., & Colford, J. M. (2003). Do U.S. Environmental Protection Agency water quality guidelines for recreational waters prevent gastrointestinal illness? A systematic review and meta-analysis. In *Environmental Health Perspectives* (Vol. 111, Issue 8, pp. 1102–1109). Public Health Services, US Dept of Health and Human Services. https://doi.org/10.1289/ehp.6241
